# Frailty, social disconnection, metabolomic signatures, and incident peripheral artery disease in type 2 diabetes: a prospective cohort study

**DOI:** 10.3389/fnut.2026.1909900

**Published:** 2026-08-21

**Authors:** Li Zhao, Xinrui Xue, Tianjiao Li, Chunyan Tang, Shuang Li, Zhen Tan, Wei Yan, Mao Ye, Xiaoping Li, Lei Liu, Hongqiang Ren

**Affiliations:** 1 Emergency Intensive Care Unit, Suining Central Hospital, Suining, Sichuan, China; 2 Department of Cardiology, Suining Central Hospital, Suining, Sichuan, China; 3 Department of Ultrasound, Suining Central Hospital, Suining, Sichuan, China; 4 Department of Radiology, Suining Central Hospital, Suining, Sichuan, China; 5 Department of Cardiology, Sichuan Provincial People’s Hospital, University of Electronic Science and Technology of China, Chengdu, Sichuan, China

**Keywords:** frailty, loneliness, metabolomics, peripheral artery disease, social isolation, type 2 diabetes, vascular risk

## Abstract

**Background:**

Frailty and social disconnection may increase vascular risk in type 2 diabetes mellitus (T2DM), but their associations with peripheral artery disease (PAD) and related metabolic pathways remain unclear.

**Methods:**

This secondary analysis of prospectively collected data from the UK Biobank included 22,582 participants with T2DM and without PAD at baseline. Frailty was defined using an adapted Fried phenotype, and social disconnection was assessed using measures of loneliness and social isolation. Cox proportional hazards models examined their separate and joint associations with incident PAD. Among 21,986 participants with nuclear magnetic resonance (NMR) metabolomic data, exposure-specific metabolomic risk scores (MRSs) were developed using least absolute shrinkage and selection operator Cox (LASSO-Cox) regression in a 70% training set and evaluated in a 30% validation set. Exploratory mediation analyses were performed in the validation set. Extreme gradient boosting (XGBoost) models with Shapley additive explanations (SHAP) were used to explore predictive patterns.

**Results:**

During a mean follow-up of 14.43 years, 1,873 incident PAD cases occurred. In the mutually adjusted model, loneliness [hazard ratio (HR), 1.19; 95% confidence interval (CI), 1.03–1.38], social isolation (HR, 1.24; 95% CI, 1.10–1.41), pre-frailty (HR, 1.22; 95% CI, 1.10–1.36), and frailty (HR, 2.02; 95% CI, 1.75–2.34) remained associated with incident PAD. Participants with frailty and either loneliness or social isolation had the highest PAD risk. In the validation set, the frailty-, loneliness-, and social isolation-related metabolomic risk scores were associated with incident PAD, with HRs per standard deviation increase of 1.22 (95% CI, 1.11–1.34), 1.23 (95% CI, 1.12–1.34), and 1.22 (95% CI, 1.11–1.33), respectively. Exploratory mediation analyses indicated that these scores statistically accounted for 16.6, 20.4, and 22.6% of the corresponding associations with PAD, respectively.

**Conclusion:**

Frailty, loneliness, and social isolation remained associated with incident PAD after mutual adjustment among individuals with T2DM. Training-derived metabolomic scores were associated with PAD in the validation set and statistically accounted for modest proportions of these associations. These findings support the potential relevance of physical vulnerability and social disconnection to PAD risk, while the metabolomic findings require external validation.

## 1 Introduction

Peripheral artery disease (PAD) affects more than 200 million people worldwide, is a leading cause of lower-extremity amputation, and is associated with a substantially increased risk of cardiovascular morbidity and mortality (1, 2). Individuals with type 2 diabetes mellitus (T2DM) have a two- to fourfold higher risk of developing PAD than those without diabetes, and once PAD develops, they are more likely to experience adverse limb and cardiovascular outcomes, including foot ulceration, revascularization, amputation, major adverse cardiovascular events, and death (3–5).

Frailty has increasingly been recognized as a marker of vulnerability in cardiovascular disease and has been associated with adverse outcomes such as hospitalization, disability, and mortality (6, 7). In patients with PAD or lower-extremity ischemia, frailty has also been linked to poorer prognosis, particularly higher mortality risk, and an increased risk of death or amputation in some cohorts of patients with chronic limb-threatening ischemia (8, 9).

Social disconnection is another important but often overlooked dimension of health. It encompasses social isolation and loneliness, two related but distinct constructs. Social isolation refers to an objective lack of social contacts or participation, whereas loneliness reflects the subjective perception of inadequate or unsatisfactory social relationships. Growing evidence has linked both social isolation and loneliness to cardiovascular disease, metabolic disorders, mortality, and diabetes-related complications (10–13).

Frailty and social disconnection may be closely interrelated. Longitudinal evidence suggests that loneliness and social isolation are associated with an increased risk of subsequent frailty, while frailty may in turn contribute to later social isolation and loneliness (14, 15). Taken together, these findings suggest that frailty and social disconnection may be relevant to PAD in individuals with T2DM, yet their individual and combined associations remain unclear.

In this study, we investigated the associations of loneliness, social isolation, and frailty with incident PAD among individuals with T2DM. We further evaluated the joint associations and interactions between frailty and social disconnection, constructed exposure-specific metabolomic risk scores using nuclear magnetic resonance (NMR) metabolomics, conducted exploratory mediation analyses, and applied interpretable machine learning to explore predictive patterns.

## 2 Materials and methods

### 2.1 Study design and participants

We conducted a secondary analysis of prospectively collected data from the UK Biobank. The UK Biobank (UKB) is a large-scale prospective cohort study that recruited approximately 500,000 participants aged 40–69 years from 22 assessment centers across the United Kingdom. The baseline assessment was conducted between 2006 and 2010, followed by three follow-up assessments: the first between 2012 and 2013, the second from 2014 onward, and the third beginning in 2019. These assessments encompassed touchscreen questionnaires, verbal interviews, physical measurements, biological sampling, and linkage to electronic health records (16). The UKB was approved by the North West Multi-Centre Research Ethics Committee, and the present study was authorized under UKB Application ID: 106027.

**FIGURE 1:**
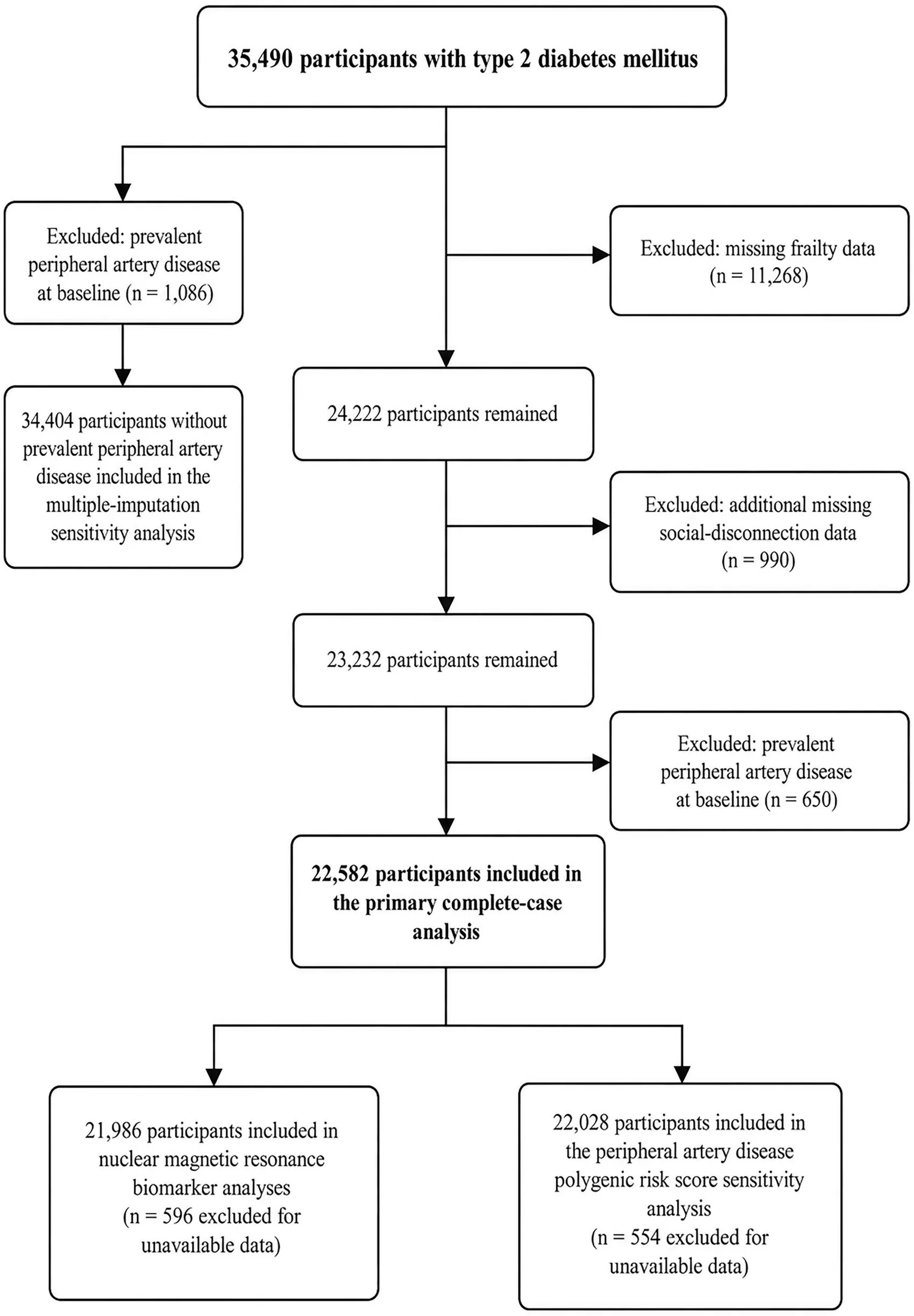
Flowchart of participant selection and analytic samples. The flowchart shows the selection of UK Biobank participants with type 2 diabetes mellitus for the primary complete-case analysis, multiple-imputation sensitivity analysis, nuclear magnetic resonance biomarker analyses, and peripheral artery disease polygenic risk score sensitivity analysis. NMR, nuclear magnetic resonance; PAD, peripheral artery disease; PRS, polygenic risk score; T2DM, type 2 diabetes mellitus.

Diabetes was defined at baseline by self-reported physician-diagnosed diabetes, current use of glucose-lowering medication, glycated hemoglobin (HbA1c) ≥ 6.5%, or blood glucose ≥ 7.0 mmol/L among participants with a recorded fasting duration of ≥ 8 h according to UK Biobank Data-Field 74 (17). Participants with possible type 1 diabetes, missing frailty or social-disconnection data, or prevalent PAD at baseline were excluded from the primary analysis. Participants without prevalent PAD were included in the missing-data sensitivity analysis, irrespective of frailty or social-disconnection data availability. The participant selection process and sample sizes for the primary, metabolomic, and genetic analyses are shown in Figure 1.

### 2.2 Definition of frailty

Frailty was assessed using an adapted Fried frailty phenotype (FP). Five FP components were considered: weight loss, exhaustion, low grip strength, low physical activity, and slow gait speed. Each component was assigned one point if present, yielding a total FP score ranging from 0 to 5. Participants with a score of 0 were classified as non-frail, those with a score of 1–2 as pre-frail, and those with a score of ≥ 3 as frail (18, 19). Detailed definitions of the five FP components are provided in Supplementary Table S1.

### 2.3 Definition of social disconnection

Social disconnection was defined by two related but distinct dimensions: loneliness and social isolation (20). Loneliness reflects the subjective perception of inadequate or unsatisfactory social relationships, whereas social isolation captures the objective scarcity of social contacts and social participation (20). Loneliness was assessed using two self-reported items: “Do you often feel lonely?” and “How often are you able to confide in someone close to you?” (21, 22). High-risk loneliness factors were defined as often feeling lonely and being able to confide in someone close less than once per month. Each high-risk item was assigned one point, yielding a loneliness score ranging from 0 to 2. Participants with a loneliness score of 2 were classified as having loneliness (21).

Social isolation was assessed using three self-reported items: household size, frequency of visits to or from friends or family, and participation in leisure or social activities (21, 22). High-risk social isolation factors were defined as living alone, visiting friends or family less than once per month, and reporting no weekly participation in any of the listed leisure or social activities. Each high-risk item was assigned one point, yielding a social isolation score ranging from 0 to 3. Participants with a social isolation score of 2 or higher were classified as having social isolation (21).

### 2.4 Definitions of outcomes

The primary outcome was incident PAD. Incident PAD was defined as the first occurrence of PAD after baseline among participants without prevalent PAD at baseline. PAD cases were identified through linkage to hospital inpatient records and death registry data. Consistent with a previous UK Biobank study of incident PAD among individuals with T2DM (4), PAD was ascertained from linked hospital inpatient and death registry records using International Classification of Diseases, Tenth Revision (ICD-10), diagnosis codes and from hospital inpatient procedure records using Office of Population Censuses and Surveys Classification of Interventions and Procedures, version 4 (OPCS-4), codes. Participants with a qualifying PAD diagnosis or procedure recorded before or at baseline were classified as having prevalent PAD and excluded from the main analysis. The complete ICD-10 and OPCS-4 code lists, presented using conventional punctuation, are provided in Supplementary Table S2. Follow-up time was calculated from the baseline assessment date to the first occurrence of PAD, death, loss to follow-up, or the end of follow-up, whichever occurred first.

### 2.5 Calculation of polygenic risk score for peripheral artery disease

The PAD polygenic risk score (PRS) was constructed using summary statistics from a published genome-wide association study of PAD (23). Single-nucleotide polymorphisms were harmonized between the discovery genome-wide association study and the UK Biobank genotype data according to chromosome, position, effect allele, and non-effect allele (24). Ambiguous or poorly imputed variants were excluded. For each participant, the PAD PRS was calculated as the weighted sum of PAD risk alleles, with the weight for each variant defined by the corresponding effect estimate from the discovery genome-wide association study (24). The score was then standardized to a mean of 0 and a standard deviation of 1 before analysis. Participants were also categorized into low, intermediate, and high genetic risk groups according to tertiles of the standardized PRS.

### 2.6 Metabolomic biomarkers

High-throughput nuclear magnetic resonance (NMR) spectroscopy-based metabolomic profiling was used to quantify 249 metabolic biomarkers (168 directly measured biomarkers and 81 ratios) from ethylenediaminetetraacetic acid (EDTA) plasma samples of approximately 280,000 UK Biobank participants. Detailed descriptions of the NMR metabolomics protocol have been reported previously (25). All metabolite concentrations were transformed using the natural logarithm [ln(X + 1)] and then standardized to z-scores to facilitate comparability across metabolites.

### 2.7 Ascertainment of covariates

Baseline covariates were selected to account for potential confounding and included sociodemographic factors, physical measurements, lifestyle behaviors, clinical conditions, biochemical markers, and medication use. Sociodemographic variables included age, sex, ethnicity, educational attainment, and the Townsend Deprivation Index (TDI). The TDI, an area-level measure of socioeconomic deprivation derived from participants’ residential postcodes, was included to capture regional socioeconomic differences (26). Educational attainment was categorized according to whether participants had obtained a college or university degree. Physical measurements included body mass index (BMI), mean arterial pressure (MAP), and pulse rate. BMI was calculated as weight (kg) divided by height squared (m^2^). Lifestyle factors included smoking status, alcohol consumption, dietary pattern, physical activity, and sleep duration. Smoking status and alcohol consumption were self-reported and categorized as never, previous, or current. Dietary pattern was classified as healthy or unhealthy according to the intake of fruits, vegetables, fish, processed meat, and unprocessed red meat, as detailed in Supplementary Table S3. Clinical and laboratory covariates included hypertension, glucose (GLU), low-density lipoprotein cholesterol (LDL-C), glycated hemoglobin (HbA1c), serum creatinine (Scr), cystatin C (Cys-C), and estimated glomerular filtration rate (eGFR). Hypertension was defined as a hospital diagnosis of hypertension, current use of antihypertensive medication, systolic blood pressure ≥ 140 mmHg, or diastolic blood pressure ≥ 90 mmHg (27). Biochemical markers were measured using standardized blood assays. The eGFR was calculated using the Chronic Kidney Disease Epidemiology Collaboration (CKD-EPI) equations (28). Medication use included insulin, aspirin, antihypertensive agents, and lipid-lowering medications.

### 2.8 Statistical analysis

#### 2.8.1 Description of the basic characteristics

All analyses were performed with R software (version 4.3.0; Institute of Statistics and Mathematics, University of Vienna, Austria). The distribution of continuous variables was evaluated using the Anderson–Darling test. Variables with an approximately normal distribution are presented as mean ± standard deviation (SD), whereas skewed variables are reported as median and interquartile range (IQR). Group differences were examined using one-way analysis of variance for normally distributed variables and the Kruskal–Wallis test for non-normally distributed variables when comparing three or more groups. Categorical variables are expressed as numbers and percentages, and between-group differences were assessed using Pearson’s chi-square test or Fisher’s exact test when expected cell counts were less than 5. Participants with missing frailty or social-disconnection data were excluded from the primary analysis. Missing covariate values were imputed using multiple imputation by chained equations, and estimates were pooled using Rubin’s rules.

#### 2.8.2 Association analysis

Cox proportional hazards models were used to examine the associations of loneliness, social isolation, and FP with the risk of incident PAD. For the primary Cox proportional hazards models, the proportional hazards assumption was evaluated using tests based on scaled Schoenfeld residuals. The results are presented as hazard ratios (HRs) with 95% confidence intervals (95% CIs). The multivariable-adjusted model included age, sex, ethnicity, TDI, educational attainment, smoking status, alcohol consumption, BMI, MAP, lipid-lowering medication, insulin use, aspirin use, antihypertensive medication, LDL-C, HbA1c, eGFR, and dietary pattern.

Multivariable-adjusted population attributable fractions (PAFs) and 95% CIs were further calculated for loneliness, social isolation, and each of the five FP components to estimate their potential contribution to incident PAD. PAFs were interpreted as the estimated proportion of incident PAD cases attributable to each factor under a causal assumption, and therefore should be interpreted cautiously in this observational study.

To account for the overlap among frailty, loneliness, and social isolation, an additional Cox model included all three exposures simultaneously, together with the covariates used in the main multivariable-adjusted model. Collinearity was assessed using generalized variance inflation factors (GVIFs), with GVIF^(1/(2 × df)) reported for variables with more than one degree of freedom.

#### 2.8.3 Joint analysis and interaction assessment

All analyses in this section were performed using the multivariable-adjusted models described above. Modified Poisson regression models with robust error variance were used to examine the associations of FP with loneliness and social isolation, separately, with results reported as prevalence ratios (PRs) and 95% CIs. Ordinal logistic regression models were used to assess the associations of loneliness and social isolation with ordered FP status, categorized as non-frail, pre-frail, and frail, with results expressed as odds ratios (ORs) and 95% CIs. The proportional odds assumption was evaluated for ordinal logistic regression models.

For the joint analysis, participants were cross-classified by FP status and loneliness or social isolation status, separately. Cox proportional hazards models were used to estimate HRs and 95% CIs for incident PAD across the joint-exposure groups, using non-frail participants without loneliness or social isolation as the reference group. Incidence rates (IRs) and 95% CIs were calculated for each group. Multiplicative interactions between FP status and loneliness or social isolation were tested by adding product terms to the Cox models. Additive interactions were assessed using the relative excess risk due to interaction (RERI) and the attributable proportion due to interaction (AP), with corresponding 95% CIs.

#### 2.8.4 Subgroup and sensitivity analyses

Subgroup analyses were performed to examine the consistency of the associations across clinically relevant strata, including age (<60 vs. ≥ 60 years), sex (male vs. female), estimated glomerular filtration rate ( < 60 vs. ≥ 60 mL/min/1.73 m^2^), TDI ( < median vs. ≥ median), obesity status (BMI < 30 vs. ≥ 30 kg/m^2^), hypertension status (yes vs. no), educational attainment (below college/university degree vs. college/university degree or above), aspirin use (yes vs. no), insulin use (yes vs. no), smoking status (yes vs. no), and alcohol consumption status (yes vs. no). The same multivariable-adjusted models were fitted within each subgroup, with the stratification variable omitted from the corresponding model. Interactions were tested by adding cross-product terms between each exposure and the subgroup variable.

Sensitivity analyses were conducted to assess the robustness of the findings. First, among participants with available PAD polygenic risk score data, the PAD polygenic risk score was additionally included in the multivariable-adjusted models to assess whether the observed associations and interactions were independent of genetic susceptibility to PAD. Second, the main analyses were repeated using Fine–Gray subdistribution hazard models, with death before incident PAD treated as a competing event. Third, because fasting insulin was unavailable, we performed an additional sensitivity analysis using the triglyceride–glucose (TyG) index as a surrogate marker of insulin resistance. Missing triglyceride and glucose values were imputed using multiple imputation by chained equations. The imputation model included the PAD event indicator, the Nelson–Aalen estimate of the cumulative baseline hazard, the three exposures, fasting duration, and all covariates included in the primary model. Triglyceride and glucose concentrations were converted from mmol/L to mg/dL, and the TyG index was calculated within each imputed dataset as ln [triglycerides (mg/dL) × glucose (mg/dL)/2]. The TyG index and fasting duration were then added to the primary multivariable-adjusted Cox models, and estimates were pooled using Rubin’s rules.

To assess potential selection bias, baseline characteristics were compared according to frailty-data availability among participants with complete social-disconnection data. Differences were quantified using standardized mean differences (SMDs), and PAD incidence was compared after excluding participants with prevalent PAD. Component-specific missingness rates and data sources are presented in Supplementary Table S4. As a further sensitivity analysis, the five frailty components and individual loneliness and social-isolation items were imputed among all 34,404 participants without prevalent PAD, assuming that data were missing at random. The imputation model included the PAD event indicator, the Nelson–Aalen estimate of the cumulative baseline hazard, and all covariates. Exposure scores were recalculated in each imputed dataset, and Cox model estimates were pooled using Rubin’s rules.

To reduce potential reverse causation due to subclinical PAD at baseline, we additionally conducted 2- and 5-year landmark sensitivity analyses. Participants who developed PAD or were censored before the corresponding landmark were excluded, and follow-up began at 2 or 5 years after baseline. The primary multivariable-adjusted Cox models were then refitted among participants who remained alive, under follow-up, and free of PAD at each landmark.

#### 2.8.5 Metabolomic biomarker selection and exploratory mediation analysis

Participants with metabolomic data (*n* = 21,986) were randomly divided into a training set (70%; *n* = 15,390) and a validation set (30%; *n* = 6,596), stratified by incident peripheral artery disease (PAD) status using a fixed random seed of 42. Baseline characteristics of the two sets are presented in Supplementary Table S5. All biomarker screening and metabolomic risk score (MRS) coefficient estimation were restricted to the training set, with no further selection or refitting in the validation set.

Among 249 nuclear magnetic resonance biomarkers, multivariable linear regression was used to identify biomarkers associated with frailty phenotype score, loneliness, and social isolation. Exposure-related biomarkers were then examined for associations with incident PAD using Cox proportional hazards models adjusted for the covariates in the main analysis. The Benjamini–Hochberg procedure was applied at both stages, with q < 0.05 considered significant. Full estimates are provided in Supplementary Data 1, 2.

Biomarkers associated with both the corresponding exposure and PAD were entered into LASSO-Cox regression. Ten-fold cross-validation was performed using partial-likelihood deviance, and the penalty parameter was selected using the one-standard-error rule. Biomarkers with nonzero coefficients were retained. Exposure-specific MRSs were calculated as weighted sums of the selected standardized biomarkers, using the training-set LASSO-Cox coefficients as weights (Supplementary Table S6).

The fixed coefficients were applied directly to the validation set. Associations between each standardized MRS and incident PAD were assessed using Cox proportional hazards models, restricted cubic splines, and adjusted cumulative PAD event curves (Supplementary Tables S7, S8). Time-dependent areas under the receiver operating characteristic curves (AUCs) were calculated at 5, 10, and 15 years (Supplementary Table S9).

Exploratory mediation analyses were conducted in the validation set. The mediator model was a multivariable linear regression model with the standardized MRS as the dependent variable. Time to incident PAD was modeled using a parametric accelerated failure-time model with a Weibull distribution fitted using the survreg function. Both models were adjusted for the covariates used in the main analysis, and an exposure–MRS interaction term was included in the outcome model. Death, loss to follow-up, and the end of follow-up were treated as censoring events; competing mortality was not modeled separately.

Average causal mediation effects and average direct effects were estimated under the reference and exposed conditions and then averaged. Non-proportion estimates were expressed on the survival-time scale returned by the accelerated failure-time mediation procedure, with negative values indicating a shorter time to incident PAD. Time ratios were obtained by exponentiating the accelerated failure-time model coefficients, with values below 1 indicating a shorter event-free time. The average proportion statistically accounted for was calculated as the average indirect effect divided by the total effect. Estimates and 95% CIs were obtained from 1,000 quasi-Bayesian Monte Carlo simulations using the R package mediation (Supplementary Table S10).

E-values were calculated to assess the sensitivity of the MRS–PAD associations to potential unmeasured confounding. Because the cumulative incidence of PAD was below 15%, adjusted hazard ratios per 1-SD increase in each MRS were treated as approximations of risk ratios. E-values were calculated for the point estimate and the confidence interval limit closest to the null (Supplementary Table S11). These analyses evaluated the MRS–PAD associations rather than the indirect effects.

Causal interpretation would require consistency, positivity, correct model specification, no unmeasured exposure–mediator, exposure–outcome, or mediator–outcome confounding, no exposure-induced mediator–outcome confounder, and conditionally independent censoring. Because the exposures and metabolomic biomarkers were assessed at or near baseline, temporal ordering and these assumptions could not be fully verified. The findings were therefore interpreted as exploratory statistical decomposition rather than evidence of causal mediation. The assumed causal structure is shown in Supplementary Figure S1.

#### 2.8.6 Exploratory XGBoost and SHAP analysis

Two exploratory binary extreme gradient boosting (XGBoost) models were fitted to examine feature contributions to 10-year PAD status. The phenotype-based model included smoking status, eGFR, HbA1c, LDL-C, MAP, BMI, educational attainment, TDI, healthy diet, sleep duration, PAD PRS, frailty score, loneliness scale, and social isolation scale. The MRS-based model replaced the three phenotype measures with their corresponding MRSs. Age, sex, and ethnicity were excluded a priori to focus on non-demographic features. Participants who developed PAD within 10 years were classified as cases, whereas those who remained PAD-free with at least 10 years of follow-up were classified as non-cases; participants censored before 10 years without PAD were excluded. Prespecified XGBoost parameters were a learning rate of 0.05, maximum tree depth of 5, subsample and column-subsample ratios of 0.8, 100 boosting rounds, and L1 and L2 regularization parameters of 0.1 and 1.0, respectively. Shapley additive explanations (SHAP) were used to assess global feature contributions and visualize fitted nonlinear and interaction patterns. The analysis was intended for exploratory pattern identification rather than clinical prediction-model development or validation.

## 3 Results

### 3.1 Participant characteristics

After excluding participants with possible type 1 diabetes, 35,490 individuals with T2DM were identified. Of these, 11,268 were excluded because of missing frailty data, 990 additional participants because of missing social-disconnection data, and 650 because of prevalent PAD at baseline, leaving 22,582 participants for the primary analysis. The missing-data sensitivity analysis included all 34,404 participants without prevalent PAD at baseline. Among participants in the primary analysis, 21,986 had available nuclear magnetic resonance metabolomic data and 22,028 had available PAD polygenic risk score data (Figure 1).

During a mean follow-up of 14.43 years, 1,873 participants developed incident PAD. Overall, 7,573 participants were non-frail, 12,431 were pre-frail, and 2,578 were frail. The mean age was 59.29 ± 7.21 years, 61.69% were women, and 90.39% were White. Frail participants were more likely to be lonely or socially isolated and generally had less favorable socioeconomic, lifestyle, and clinical profiles. They also had higher body mass index and cystatin C levels, lower estimated glomerular filtration rate, and greater medication use. Blood glucose and HbA1c levels were similar across frailty groups (Table 1).

**TABLE 1:** Baseline characteristics according to frailty phenotype.

Characteristic	Overall (*n* = 22,582)	Non-frail (*n* = 7,573)	Pre-frail (*n* = 12,431)	Frail (*n* = 2,578)	*P*-value	SMD
Demographic characteristics						
Age, years	59.289 ± 7.212	59.459 ± 7.204	59.17 ± 7.264	59.364 ± 6.976	0.02	0.027
Sex, n (%)					< 0.001	0.132
Female	13,931 (61.69)	4,833 (63.82)	7,703 (61.97)	1,395 (54.11)		
Male	8,651 (38.31)	2,740 (36.18)	4,728 (38.03)	1,183 (45.89)		
Ethnicity, n (%)					< 0.001	0.138
Other	2,171 (9.61)	539 (7.12)	1,288 (10.36)	344 (13.34)		
White	20,411 (90.39)	7,034 (92.88)	11,143 (89.64)	2,234 (86.66)		
Educational attainment, n (%)					< 0.001	0.183
Other	15,999 (71.29)	5,109 (67.83)	8,846 (71.64)	2,044 (79.81)		
College/university or above	6,442 (28.71)	2,423 (32.17)	3,502 (28.36)	517 (20.19)		
Townsend deprivation index	−1.67 (−3.4 to 1.36)	−2.19 (−3.64 to 0.38)	−1.6 (−3.35 to 1.53)	−0.11 (−2.6 to 3.08)	< 0.001	0.338
Lifestyle factors						
Smoking status, n (%)					< 0.001	0.135
Never	10,494 (46.63)	3,630 (48.07)	5,741 (46.34)	1,123 (43.82)		
Previous	9,653 (42.90)	3,265 (43.24)	5,336 (43.07)	1,052 (41.05)		
Current	2,356 (10.47)	656 (8.69)	1,312 (10.59)	388 (15.14)		
Alcohol consumption, n (%)					< 0.001	0.274
Never	1,429 (6.33)	314 (4.15)	840 (6.76)	275 (10.68)		
Previous	1,418 (6.28)	304 (4.02)	806 (6.49)	308 (11.96)		
Current	19,721 (87.38)	6,952 (91.84)	10,776 (86.75)	1,993 (77.37)		
Sleep duration, n (%)					< 0.001	0.359
< 7 h	5,949 (26.43)	1,669 (22.07)	3,359 (27.10)	921 (36.10)		
7–9 h	13,885 (61.69)	5,179 (68.50)	7,609 (61.38)	1,097 (43.00)		
> 9 h	2,674 (11.88)	713 (9.43)	1,428 (11.52)	533 (20.89)		
Healthy diet, n (%)					< 0.001	0.135
No	11,280 (49.98)	3,536 (46.72)	6,279 (50.54)	1,465 (56.83)		
Yes	11,290 (50.02)	4,033 (53.28)	6,144 (49.46)	1,113 (43.17)		
Loneliness score, n (%)					< 0.001	0.373
0	14,062 (62.27)	5,294 (69.91)	7,614 (61.25)	1,154 (44.76)		
1	6,532 (28.93)	1,864 (24.61)	3,698 (29.75)	970 (37.63)		
2	1,988 (8.80)	415 (5.48)	1,119 (9.00)	454 (17.61)		
Social isolation score, n (%)					< 0.001	0.307
0	10,577 (46.84)	4,034 (53.27)	5,680 (45.69)	863 (33.48)		
1	9,203 (40.75)	2,866 (37.84)	5,155 (41.47)	1,182 (45.85)		
2	2,531 (11.21)	619 (8.17)	1,449 (11.66)	463 (17.96)		
3	271 (1.20)	54 (0.71)	147 (1.18)	70 (2.72)		
Frailty phenotype components, n (%)						
Low grip strength	5,542 (24.54)	0 (0.00)	3,696 (29.73)	1,846 (71.61)	–	–
Low physical activity	6,110 (27.06)	0 (0.00)	4,179 (33.62)	1,931 (74.90)	–	–
Self-reported weight loss	5,576 (24.69)	0 (0.00)	4,406 (35.44)	1,170 (45.38)	–	–
Exhaustion	3,951 (17.50)	0 (0.00)	2,365 (19.03)	1,586 (61.52)	–	–
Slow walking pace	4,085 (18.09)	0 (0.00)	2,050 (16.49)	2,035 (78.94)	–	–
Clinical and laboratory variables						
BMI, kg/m^2^	30.036 (26.76–34.05)	28.47 (25.669–32.03)	30.492 (27.236–34.369)	33.061 (29.378–37.843)	< 0.001	0.547
Mean arterial pressure, mmHg	101.88 ± 11.258	102.825 ± 11.271	101.664 ± 11.121	100.13 ± 11.617	< 0.001	0.158
Pulse rate, beats/min	74.339 ± 13.063	73.555 ± 12.759	74.403 ± 13.064	76.35 ± 13.714	< 0.001	0.141
Hypertension, n (%)					< 0.001	0.161
No	4,671 (20.68)	1,738 (22.95)	2,581 (20.76)	352 (13.65)		
Yes	17,911 (79.32)	5,835 (77.05)	9,850 (79.24)	2,226 (86.35)		
Glucose, mmol/L	7.088 (5.545–8.602)	7.176 (5.752–8.456)	7.042 (5.476–8.65)	6.851 (5.388–8.951)	0.155	0.03
LDL-C, mmol/L	2.785 (2.281–3.462)	2.888 (2.339–3.601)	2.758 (2.267–3.426)	2.654 (2.192–3.239)	< 0.001	0.178
HbA1c, %	6.579 (5.892–7.366)	6.505 (5.755–7.237)	6.606 (5.938–7.393)	6.698 (6.085–7.613)	0.599	0.015
Serum creatinine, μmol/L	72.1 (62–83.2)	72.7 (63.3–83.75)	71.9 (61.8–82.8)	70.8 (59.6–84.4)	< 0.001	0.07
Cystatin C, mg/L	0.924 (0.83–1.043)	0.898 (0.815–1.004)	0.929 (0.834–1.047)	1 (0.879–1.17)	< 0.001	0.369
eGFR, mL/min/1.73 m^2^	88.19 (77.161–98.439)	89.78 (79.599–99.218)	88.059 (77.129–98.479)	83.188 (69.285–95.241)	< 0.001	0.297
Medication use						
Lipid-lowering medication, n (%)					< 0.001	0.241
No	8,669 (38.39)	3,378 (44.61)	4,584 (36.88)	707 (27.45)		
Yes	13,910 (61.61)	4,195 (55.39)	7,846 (63.12)	1,869 (72.55)		
Antihypertensive medication, n (%)					< 0.001	0.293
No	10,211 (45.22)	3,968 (52.40)	5,442 (43.78)	801 (31.09)		
Yes	12,368 (54.78)	3,605 (47.60)	6,988 (56.22)	1,775 (68.91)		
Insulin use, n (%)					< 0.001	0.122
No	19,119 (84.68)	6,474 (85.49)	10,623 (85.46)	2,022 (78.49)		
Yes	3,460 (15.32)	1,099 (14.51)	1,807 (14.54)	554 (21.51)		
Aspirin use, n (%)					< 0.001	0.126
No	13,319 (58.99)	4,786 (63.20)	7,143 (57.47)	1,390 (53.94)		
Yes	9,261 (41.01)	2,787 (36.80)	5,287 (42.53)	1,187 (46.06)		

Values are presented as mean ± standard deviation (SD), median (interquartile range [IQR]), or n (%), as appropriate. Percentages are column percentages. Descriptive statistics were based on observed data, whereas missing covariate values were imputed using multiple imputation by chained equations before regression analyses. Frailty phenotype was categorized as non-frail (score = 0), pre-frail (score = 1–2), and frail (score ≥ 3). Frailty phenotype components are presented as the number and percentage of participants meeting each criterion. Because these components were used to define the frailty categories, between-group *P*-values and standardized mean differences were not calculated. *P*-values were obtained using one-way analysis of variance, the Kruskal–Wallis test, Pearson’s chi-square test, or Fisher’s exact test, as appropriate. ANOVA, analysis of variance; BMI, body mass index; eGFR, estimated glomerular filtration rate; HbA1c, glycated hemoglobin; IQR, interquartile range; LDL-C, low-density lipoprotein cholesterol; MAP, mean arterial pressure; SD, standard deviation; SMD, standardized mean difference; TDI, Townsend Deprivation Index.

Among 32,838 participants with complete social-disconnection data, 9,606 had missing frailty data. Differences according to frailty-data availability were generally modest: all absolute standardized mean differences were below 0.25 and most were below 0.10. Participants with missing frailty data were slightly older, more often men and current smokers, and had greater socioeconomic deprivation, higher BMI, and lower estimated glomerular filtration rate (Supplementary Table S12). Among participants without prevalent PAD, incident PAD occurred in 9.15% of those with missing frailty data and 8.29% of those with complete frailty data (Supplementary Table S13).

Missingness was highest for low physical activity (15.8%), followed by exhaustion (7.4%), low grip strength (3.3%), weight loss (3.1%), and slow gait speed (2.6%) (Supplementary Table S4).

### 3.2 Loneliness and social isolation scales, frailty, and risk of PAD among individuals with T2DM

No evidence of violation of the proportional hazards assumption was observed for frailty phenotype, frailty score, loneliness, loneliness score, social isolation, or social isolation score, with Schoenfeld residual test *P*-values of 0.190, 0.069, 0.947, 0.901, 0.217, and 0.133, respectively. In the multivariable-adjusted Cox models, loneliness was associated with a higher risk of incident PAD compared with no loneliness (HR, 1.25; 95% CI, 1.08–1.45; *P* = 0.002) (Table 2). Each one-point increase in the loneliness score was also associated with an increased risk of PAD (HR, 1.15; 95% CI, 1.08–1.23; *P* < 0.001).

**TABLE 2:** Associations of loneliness, social isolation, and frailty with incident PAD among individuals with T2DM.

Exposure	Cases/total n (%)	Unadjusted HR (95% CI)	*P*-value	Multivariable-adjusted HR (95% CI)	*P*-value
Loneliness scale					
No loneliness	1,662/20,594 (8.07)	Reference		Reference	
Loneliness	211/1,988 (10.61)	1.36 (1.18–1.57)	< 0.001	1.25 (1.08–1.45)	0.002
Per one-point increase	1,873/22,582 (8.29)	1.21 (1.14–1.30)	< 0.001	1.15 (1.08–1.23)	< 0.001
Social isolation scale					
No social isolation	1,572/19,780 (7.95)	Reference		Reference	
Social isolation	301/2,802 (10.74)	1.44 (1.27–1.62)	< 0.001	1.25 (1.10–1.42)	0.001
Per one–point increase	1,873/22,582 (8.29)	1.22 (1.15–1.29)	< 0.001	1.14 (1.07–1.21)	< 0.001
Frailty scale					
Non-frail	501/7,573 (6.62)	Reference		Reference	
Pre-frail	1,021/12,431 (8.21)	1.28 (1.15–1.42)	< 0.001	1.22 (1.09–1.36)	< 0.001
Frail	351/2,578 (13.62)	2.37 (2.06–2.71)	< 0.001	2.01 (1.74–2.33)	< 0.001
Per one-point increase	1,873/22,582 (8.29)	1.31 (1.26–1.36)	< 0.001	1.26 (1.20–1.31)	< 0.001

CI, confidence interval; HR, hazard ratio; PAD, peripheral artery disease; T2DM, type 2 diabetes mellitus. The multivariable-adjusted model included age, sex, ethnicity, Townsend Deprivation Index, educational attainment, smoking status, alcohol consumption, body mass index, mean arterial pressure, lipid-lowering medication, insulin use, aspirin use, antihypertensive medication, LDL-C, HbA1c, eGFR, and dietary pattern.

Similarly, social isolation was significantly associated with incident PAD. Compared with participants without social isolation, those with social isolation had a higher risk of PAD after multivariable adjustment (HR, 1.25; 95% CI, 1.10–1.42; *P* = 0.001). Each one-point increase in the social isolation score was associated with a 14% higher risk of PAD (HR, 1.14; 95% CI, 1.07–1.21; *P* < 0.001).

Frailty showed a graded association with incident PAD. Compared with non-frail participants, pre-frail participants had a moderately increased risk of PAD (HR, 1.22; 95% CI, 1.09–1.36; *P* < 0.001), whereas frail participants had an approximately twofold higher risk (HR, 2.01; 95% CI, 1.74–2.33; *P* < 0.001). Each one-point increase in the frailty phenotype score was associated with a higher risk of PAD (HR, 1.26; 95% CI, 1.20–1.31; *P* < 0.001).

When frailty, loneliness, and social isolation were included simultaneously, loneliness (HR, 1.19; 95% CI, 1.03–1.38; *P* = 0.019), social isolation (HR, 1.24; 95% CI, 1.10–1.41; *P* = 0.001), pre-frailty (HR, 1.22; 95% CI, 1.10–1.36; *P* < 0.001), and frailty (HR, 2.02; 95% CI, 1.75–2.34; *P* < 0.001) remained associated with incident PAD. The association with loneliness was modestly attenuated, whereas the associations of social isolation and frailty were largely unchanged (Supplementary Table S14). No substantial collinearity was observed; all adjusted GVIF values were ≤ 1.21 (Supplementary Table S15).

**FIGURE 2:**
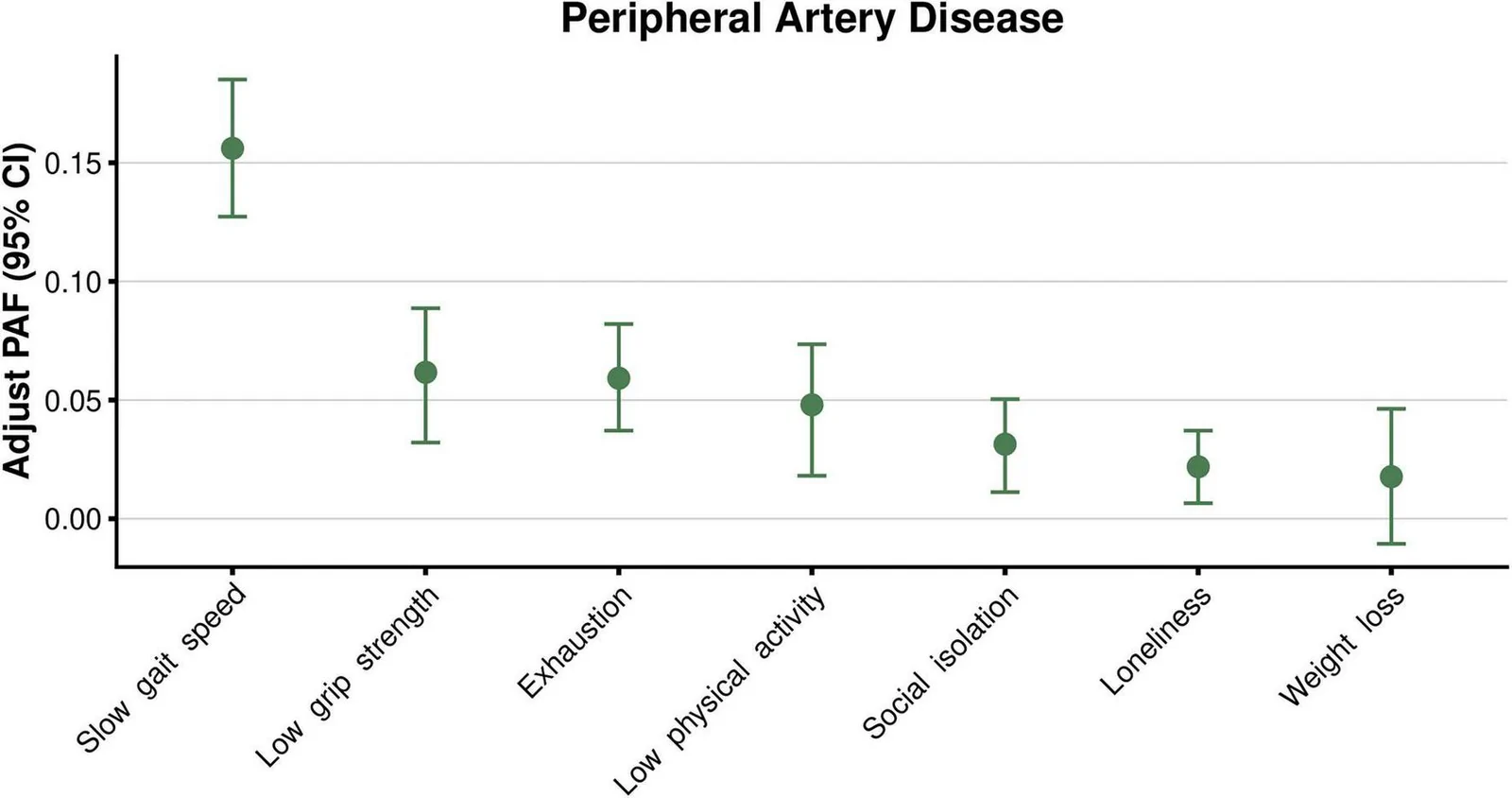
Adjusted population attributable fractions for incident peripheral artery disease. Multivariable-adjusted population attributable fractions for social disconnection indicators and individual frailty phenotype components in relation to incident peripheral artery disease. Estimates are presented with 95% confidence intervals and should be interpreted cautiously under the observational study design. CI, confidence interval; PAD, peripheral artery disease; PAF, population attributable fraction.

The associations between individual frailty components and incident PAD are presented in Supplementary Table S16. In the multivariable-adjusted PAF analysis, the frailty components and social disconnection indicators contributed differently to the burden of incident PAD (Figure 2 and Supplementary Table S17). Slow gait speed accounted for the largest estimated attributable fraction, followed by low grip strength, exhaustion, low physical activity, social isolation, and loneliness.

### 3.3 Joint analysis and interaction assessment

In the multivariable-adjusted modified Poisson regression models, frailty status was positively associated with both loneliness and social isolation. Compared with non-frail participants, the PRs for loneliness were 1.51 (95% CI, 1.35–1.69) in pre-frail participants and 2.60 (95% CI, 2.27–2.98) in frail participants. The corresponding PRs for social isolation were 1.34 (95% CI, 1.23–1.46) and 1.89 (95% CI, 1.69–2.11), respectively. In ordinal logistic regression models, loneliness and social isolation were associated with higher odds of more advanced frailty status (OR, 1.91; 95% CI, 1.74–2.10 for loneliness; OR, 1.56; 95% CI, 1.44–1.70 for social isolation) (Supplementary Table S18).

**FIGURE 3:**
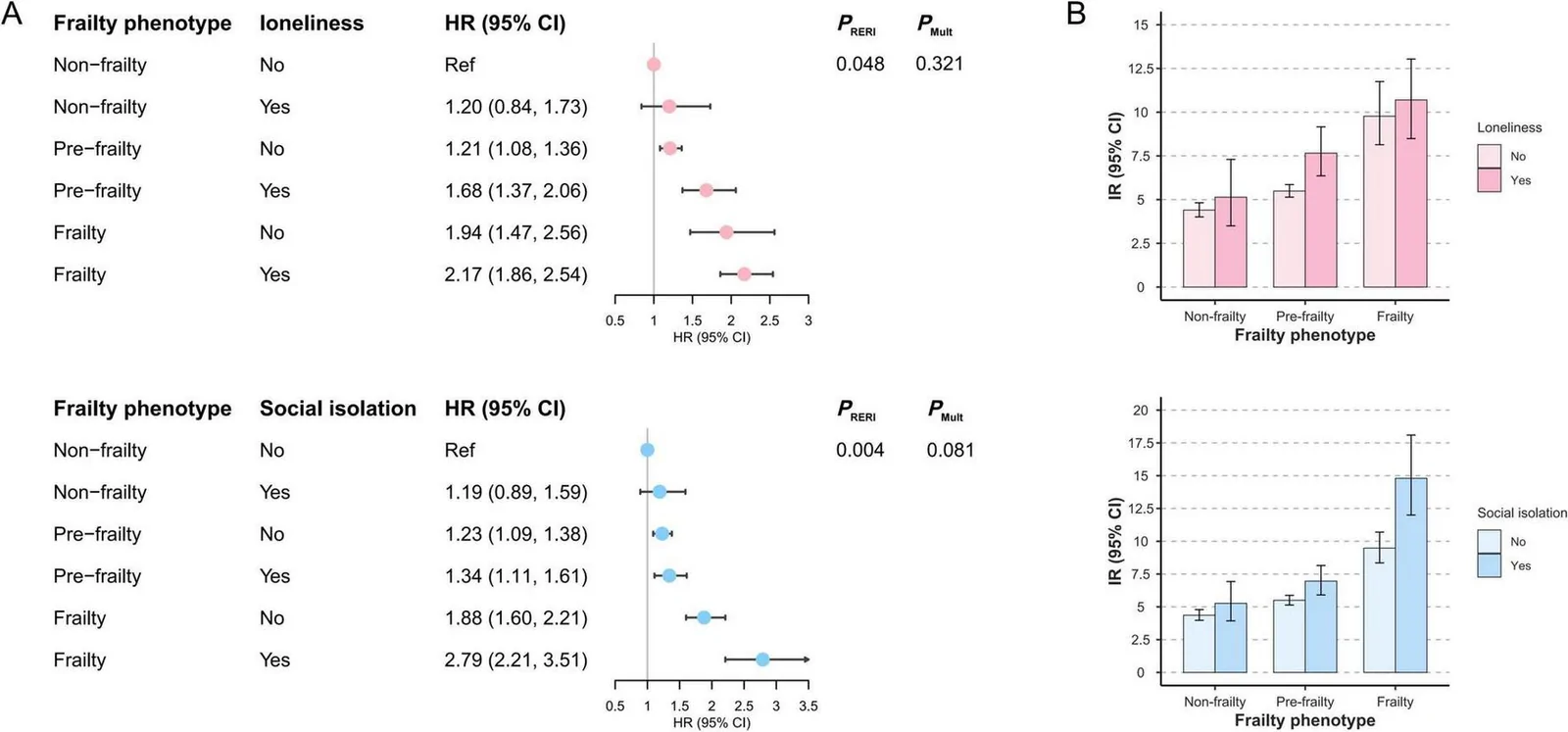
Joint associations of frailty phenotype and social disconnection with incident peripheral artery disease. **(A)** Multivariable-adjusted hazard ratios for joint categories of frailty phenotype with loneliness (upper) and social isolation (lower), using non-frail participants without the corresponding social-disconnection indicator as the reference group. **(B)** The corresponding incidence rates across joint-exposure categories. The *P*-values for additive and multiplicative interactions are also shown. Additive interaction was assessed using the relative excess risk due to interaction.

In the joint Cox regression analysis, participants with both frailty and social disconnection had the highest risk of incident PAD (Figure 3 and Supplementary Table S19). Compared with non-frail participants without loneliness, the HRs for PAD were 1.20 (95% CI, 0.84–1.73) for non-frail participants with loneliness, 1.21 (95% CI, 1.08–1.36) for pre-frail participants without loneliness, 1.68 (95% CI, 1.37–2.06) for pre-frail participants with loneliness, 1.94 (95% CI, 1.47–2.56) for frail participants without loneliness, and 2.17 (95% CI, 1.86–2.54) for frail participants with loneliness.

A similar pattern was observed for social isolation. Compared with non-frail participants without social isolation, the HRs were 1.19 (95% CI, 0.89–1.59) for non-frail participants with social isolation, 1.23 (95% CI, 1.09–1.38) for pre-frail participants without social isolation, 1.34 (95% CI, 1.11–1.61) for pre-frail participants with social isolation, 1.88 (95% CI, 1.60–2.21) for frail participants without social isolation, and 2.79 (95% CI, 2.21–3.51) for frail participants with social isolation.

Incidence rates increased across joint frailty and social disconnection categories, with the highest rates observed among frail participants with loneliness or social isolation. Additive interaction estimates, including RERI and AP, are presented in Supplementary Table S19. No clear multiplicative interaction was observed.

### 3.4 Subgroup and sensitivity analyses

Subgroup analyses showed that the associations of loneliness, social isolation, and frailty phenotype with incident PAD were generally consistent across strata defined by age, sex, obesity status, eGFR, Townsend Deprivation Index, educational attainment, hypertension status, insulin use, aspirin use, smoking status, and alcohol consumption, with no significant interactions observed (Supplementary Table S20).

In sensitivity analyses additionally adjusting for PAD polygenic risk score, the associations of loneliness, social isolation, and frailty phenotype with incident PAD remained largely unchanged, and the joint associations between frailty and social disconnection were consistent with the main findings (Supplementary Table S21). Similar results were observed in Fine–Gray competing risk models treating death before PAD as a competing event; loneliness, social isolation, and frailty phenotype remained associated with higher subdistribution hazards of incident PAD, with the highest risks observed among frail participants with loneliness or social isolation (Supplementary Table S22).

In the TyG sensitivity analysis, further adjustment for the TyG index did not materially alter the associations with incident PAD. The adjusted HRs were 1.25 (95% CI, 1.08–1.44) for loneliness, 1.25 (95% CI, 1.10–1.42) for social isolation, 1.22 (95% CI, 1.09–1.36) for pre-frailty, and 1.99 (95% CI, 1.72–2.30) for frailty. No evidence of substantial multicollinearity was observed in the collinearity diagnostics (Supplementary Table S23).

Participants excluded because of missing exposure data had a higher crude incidence of PAD than those included in the main analysis (9.15% vs. 8.29%). After adjustment, however, PAD risk did not differ significantly between the two groups (HR for included vs. excluded participants, 0.93; 95% CI, 0.86–1.01; *P* = 0.077; Supplementary Table S13).

Results were similar after imputing the exposure variables among all 34,404 participants without prevalent PAD. Loneliness (HR, 1.21; 95% CI, 1.08–1.36), social isolation (HR, 1.15; 95% CI, 1.04–1.27), pre-frailty (HR, 1.30; 95% CI, 1.19–1.42), and frailty (HR, 2.05; 95% CI, 1.82–2.30) remained associated with incident PAD (Supplementary Table S24). The joint associations of frailty with loneliness and social isolation were also consistent with the main findings (Supplementary Table S25).

The findings were largely consistent in the lagged sensitivity analyses. After excluding events occurring within the first 2 years, loneliness (HR, 1.23; 95% CI, 1.05–1.43), social isolation (HR, 1.24; 95% CI, 1.08–1.41), pre-frailty (HR, 1.17; 95% CI, 1.05–1.31), and frailty (HR, 1.85; 95% CI, 1.59–2.16) remained associated with incident PAD. After applying a 5-year lag, the associations remained evident for loneliness (HR, 1.23; 95% CI, 1.04–1.46), social isolation (HR, 1.21; 95% CI, 1.04–1.41), and frailty (HR, 1.68; 95% CI, 1.41–2.00), whereas the association with pre-frailty was attenuated (HR, 1.13; 95% CI, 1.00–1.28; P = 0.056). Associations based on the continuous loneliness, social isolation, and frailty scores also remained significant in both analyses (Supplementary Table S26).

### 3.5 Metabolomic biomarker screening and MRS construction

Among the 21,986 participants with metabolomic data, 15,390 were assigned to the training set and 6,596 to the validation set. Baseline characteristics were comparable between the two sets, with all standardized mean differences below 0.1 (Supplementary Table S5).

In the training set, multivariable linear regression identified 211, 30, and 176 NMR biomarkers associated with frailty phenotype score, loneliness, and social isolation, respectively, after Benjamini–Hochberg correction (Supplementary Data 1). Of these, 158, 30, and 144 were also associated with incident PAD in Cox models (Supplementary Data 2) and were entered into LASSO-Cox regression. Using the one-standard-error criterion, 11, 6, and 9 biomarkers were retained for the frailty-, loneliness-, and social isolation-related MRSs, respectively. Their coefficients were estimated in the training set and fixed before application to the validation set. Cross-validation curves and coefficient trajectories are shown in Supplementary Figure S2, and the selected biomarkers and coefficients are listed in Supplementary Table S6.

### 3.6 MRS associations and exploratory mediation analysis

All three MRSs were associated with incident PAD in the training set (Supplementary Table S7), with similar associations observed in the validation set (Supplementary Table S8). In the validation set, the adjusted HRs per 1-SD increase were 1.22 (95% CI, 1.11–1.34), 1.23 (95% CI, 1.12–1.34), and 1.22 (95% CI, 1.11–1.33) for the frailty-, loneliness-, and social isolation-related MRSs, respectively. The corresponding HRs for the highest versus lowest tertile were 1.46 (95% CI, 1.15–1.85), 1.61 (95% CI, 1.27–2.04), and 1.48 (95% CI, 1.17–1.88).

**FIGURE 4:**
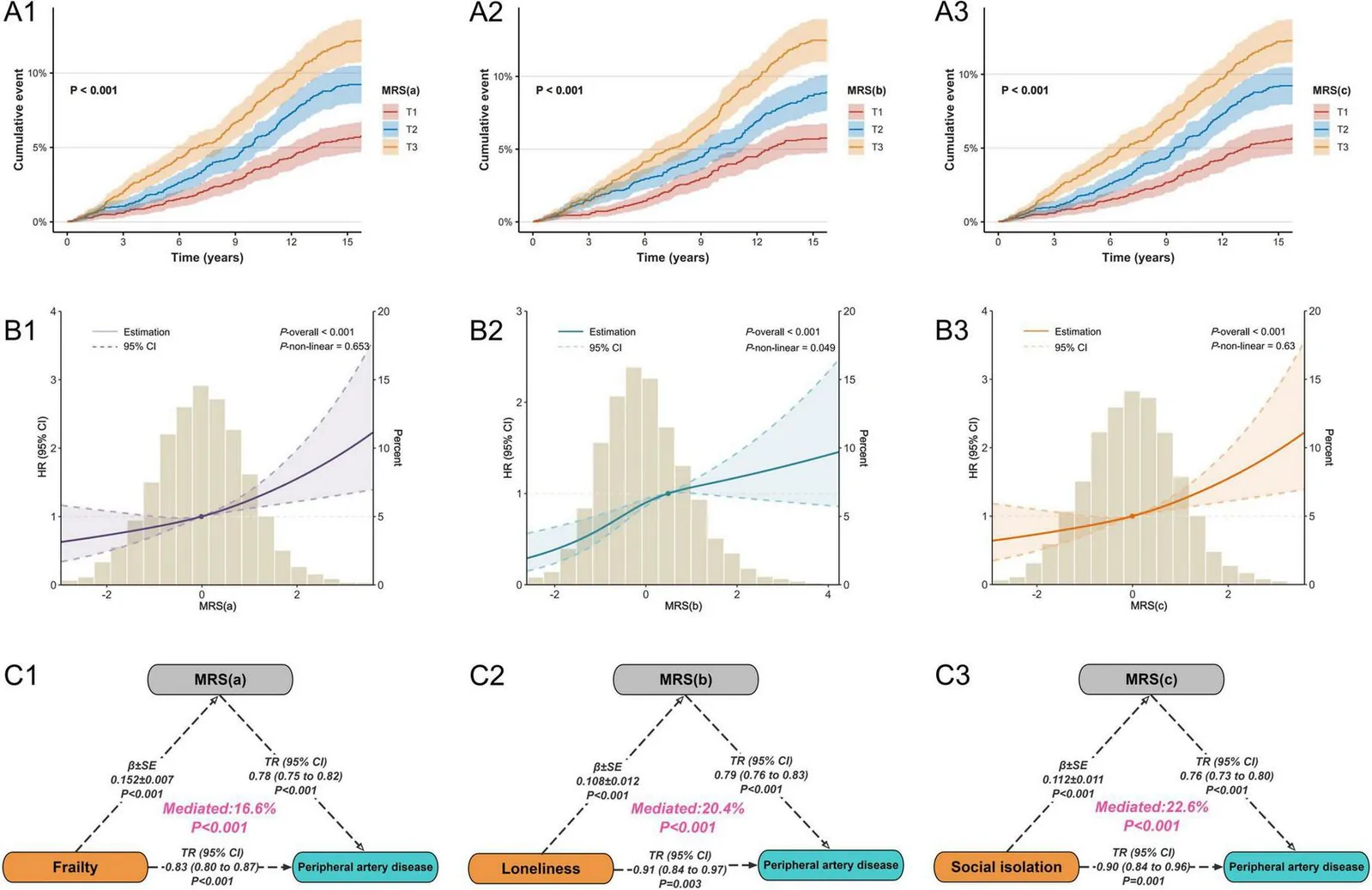
Metabolomic risk scores, incident peripheral artery disease, and exploratory mediation analyses. **(A1–A3)** Adjusted cumulative PAD event curves across tertiles of the frailty-, loneliness-, and social isolation-related MRSs, respectively. **(B1–B3)** Restricted cubic spline associations between the corresponding MRSs and incident PAD, with histograms indicating MRS distributions. **(C1–C3)** Exploratory mediation analyses for frailty phenotype score, loneliness, and social isolation through their corresponding MRSs. The mediation analyses were conducted using Weibull accelerated failure-time models in the validation set. β, regression coefficient; CI, confidence interval; HR, hazard ratio; MRS, metabolomic risk score; PAD, peripheral artery disease; SE, standard error; T1–T3, tertiles of MRS; TR, time ratio.

Restricted cubic spline analyses showed positive associations for all three MRSs. Evidence of nonlinearity was observed for the loneliness-related MRS (P for nonlinearity = 0.049), but not for the frailty- or social isolation-related MRSs. Adjusted cumulative PAD event probability increased across MRS tertiles (Figure 4). Time-dependent AUCs ranged from 0.596 to 0.620 in the training set and from 0.602 to 0.637 in the validation set (Supplementary Figures S3, S4 and Supplementary Table S9).

In exploratory mediation analyses, the frailty-, loneliness-, and social isolation-related MRSs statistically accounted for 16.6% (95% CI, 12.7–21.4%), 20.4% (95% CI, 12.1–45.2%), and 22.6% (95% CI, 14.7–43.8%) of the corresponding associations with incident PAD, respectively (all *P* < 0.001; Figure 4 and Supplementary Table S10). Negative indirect-effect estimates indicated a shorter time to incident PAD. E-values for the MRS–PAD associations ranged from 1.74 to 1.76, with corresponding confidence-limit *E*-values ranging from 1.46 to 1.49 (Supplementary Table S11). These findings were interpreted as exploratory statistical decomposition rather than causal mediation.

### 3.7 Exploratory XGBoost and SHAP analysis

**FIGURE 5:**
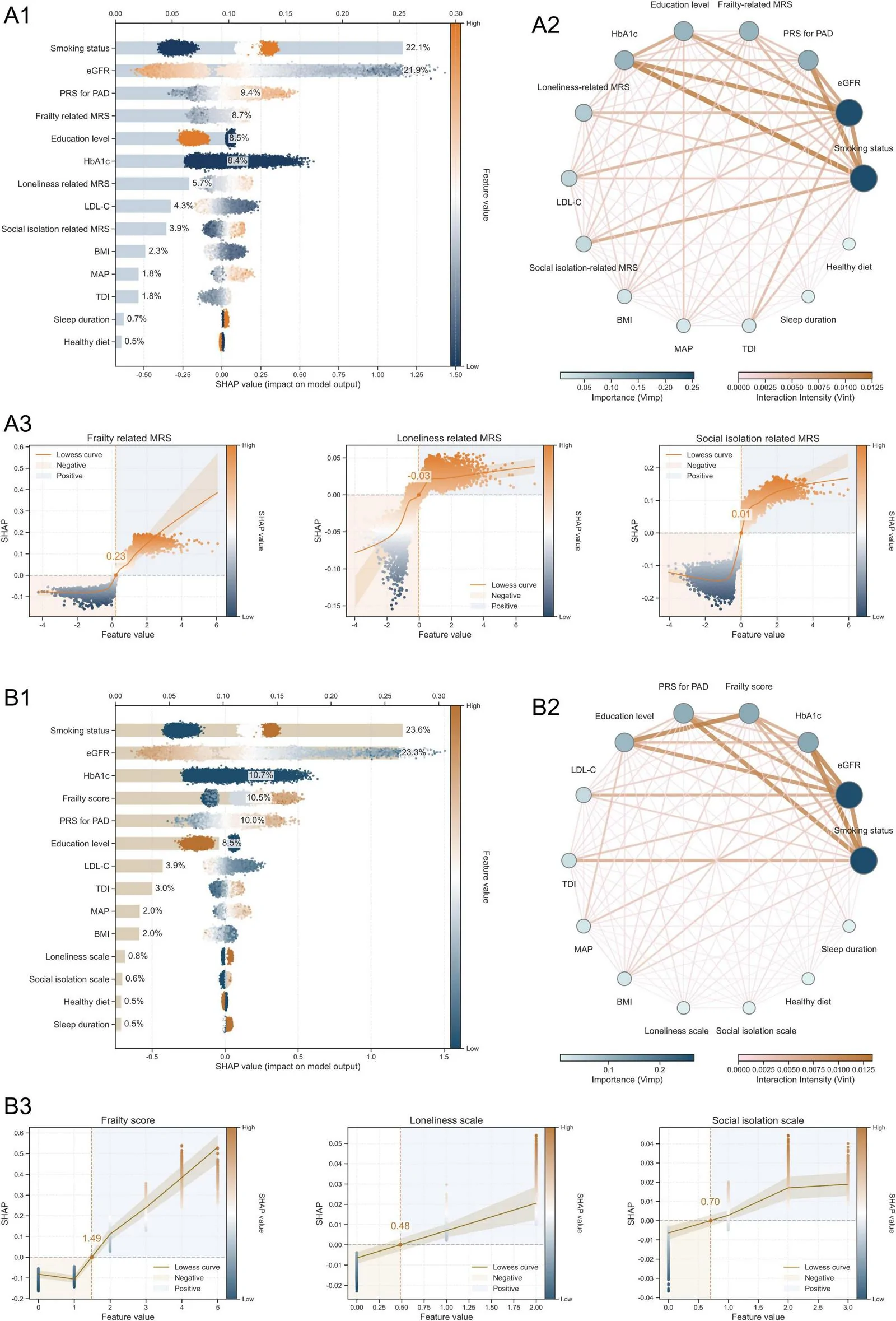
Exploratory XGBoost and SHAP analysis of 10-year PAD status. **(A)** The MRS-based model, which included baseline covariates, PAD polygenic risk score, and the frailty-, loneliness-, and social isolation-related MRSs. **(B)** The phenotype-based model, which included baseline covariates, PAD polygenic risk score, frailty score, loneliness scale, and social isolation scale. **(A1,B1)** Global SHAP feature importance, expressed as mean absolute SHAP values, together with SHAP summary plots showing the direction and magnitude of each predictor’s contribution. **(A2,B2)** SHAP interaction networks illustrating pairwise interactions among predictors. **(A3)** SHAP dependence plots for the frailty-, loneliness-, and social isolation-related MRSs. **(B3)** SHAP dependence plots for frailty score, loneliness scale, and social isolation scale. These exploratory analyses were used to visualize predictive importance, nonlinear associations, and feature interactions rather than to infer causality. BMI, body mass index; eGFR, estimated glomerular filtration rate; HbA1c, glycated hemoglobin; LDL-C, low-density lipoprotein cholesterol; MRS, metabolomic risk score; PAD, peripheral artery disease; PRS, polygenic risk score; SHAP, Shapley additive explanations; TDI, Townsend Deprivation Index.

In exploratory XGBoost analyses of 10-year PAD status, smoking status and eGFR made the largest contributions to the fitted model output in both models. In the MRS-based model, the frailty-related MRS contributed more than the loneliness- and social isolation-related MRSs. In the phenotype-based model, the frailty score contributed more than the loneliness and social isolation scores (Figure 5). SHAP main effects were generally larger than the corresponding summed interaction effects (Supplementary Figures S5, S7). The strongest pairwise interaction patterns mainly involved smoking status, eGFR, and HbA1c, with additional interactions involving the frailty score and the three MRSs (Supplementary Figures S6, S8). These analyses describe exploratory feature-attribution patterns within the prespecified non-demographic feature set and should not be interpreted as evidence of causality or validated clinical predictive performance.

## 4 Discussion

In this secondary analysis of the prospective UK Biobank cohort, loneliness, social isolation, and frailty phenotype remained associated with incident PAD after adjustment for conventional risk factors and mutual adjustment for the three exposures. Frailty showed a graded association with PAD, and participants with both frailty and social disconnection had the highest risk. The three exposure-specific MRSs were also associated with PAD in the validation set and statistically accounted for part of the corresponding associations. Exploratory XGBoost analyses further described the relative contributions of frailty, social disconnection, and their metabolomic signatures within a prespecified non-demographic feature set. These findings suggest that physical vulnerability and social disconnection represent vascular risk domains in T2DM that are not fully captured by conventional risk factors.

PAD is a common and clinically important manifestation of systemic atherosclerosis in patients with T2DM. Current risk assessment is largely centered on established cardiometabolic factors, including smoking, glycemic control, blood pressure, dyslipidemia, renal function, and genetic susceptibility. Our findings extend this framework by showing that frailty and social disconnection were associated with future PAD beyond these conventional factors. Because frailty and social disconnection are relatively easy to assess in clinical and community settings, they may help identify individuals with T2DM who have an elevated burden of vascular vulnerability not fully captured by standard laboratory or medication-based risk profiles.

The association between frailty and PAD is biologically plausible and consistent with previous evidence linking frailty to adverse cardiovascular outcomes (6–9, 29). Prior studies in patients with established PAD or chronic limb-threatening ischemia have shown that frailty is associated with poorer prognosis, including higher mortality and risk of amputation (8, 9). Our study extends these observations by showing that frailty phenotype was associated with incident PAD among individuals with T2DM who were free of PAD at baseline. This suggests that frailty may not simply be a consequence of established limb ischemia, but may also mark a preclinical state of vascular vulnerability. Potential mechanisms include chronic low-grade inflammation, endothelial dysfunction, impaired skeletal muscle metabolism, sarcopenia, reduced physical activity, autonomic dysregulation, and accelerated vascular aging (30, 31). Insulin resistance and compensatory hyperinsulinemia may further promote endothelial dysfunction, systemic inflammation, and atherogenic dyslipidemia, whereas reduced endogenous insulin secretory capacity in more advanced T2DM may reflect greater metabolic disease severity and contribute to both frailty-related vulnerability and PAD risk (32–35). Among the frailty components, slow gait speed showed the largest estimated population-attributable fraction. This finding is consistent with evidence that gait speed is a simple but informative marker of cardiovascular risk (36), and is especially relevant to PAD because walking impairment may reflect both systemic functional decline and early lower-extremity vascular insufficiency. In patients with established PAD, slower walking speed has also been associated with worse prognosis (37). Nevertheless, this result should be interpreted cautiously, because subclinical PAD before diagnosis may also contribute to gait slowing.

Our findings also support the vascular relevance of social disconnection in T2DM. Previous studies have linked loneliness and social isolation to coronary heart disease, stroke, mortality, diabetes, and diabetes-related complications (10–13, 20–22, 38). In a recent UK Biobank study of individuals with T2DM, loneliness, but not social isolation, was associated with incident cardiovascular disease (21). Our findings differ partly from that study, as both loneliness and social isolation were associated with incident PAD. This discrepancy may reflect differences in outcome definition. Broad cardiovascular disease endpoints are often driven by coronary and cerebrovascular events, whereas PAD may be more closely related to mobility limitation, physical inactivity, delayed healthcare seeking, foot care, and social support for chronic disease management. Objective social isolation may therefore be particularly relevant to PAD, a disease in which behavioral, functional, and healthcare-access pathways can influence progression from subclinical vascular disease to clinically recognized limb ischemia. At the same time, loneliness and social isolation should be interpreted as markers of broader behavioral, functional, and biological vulnerability rather than as isolated causal exposures.

Frailty and social disconnection were closely linked in our study. Pre-frail and frail participants had a higher prevalence of both loneliness and social isolation, while loneliness and social isolation were associated with more advanced frailty status. These findings are consistent with longitudinal evidence suggesting bidirectional relationships between social disconnection and frailty (14, 15). Social isolation and loneliness may accelerate frailty progression through reduced activity, poorer sleep, depressive symptoms, unhealthy behaviors, and limited access to care; conversely, frailty may restrict mobility and social participation, thereby increasing isolation and loneliness. In the joint analysis, participants with both frailty and social disconnection had the highest incidence rates and PAD risk. Although no clear multiplicative interaction was observed, the absolute risk accumulation remained clinically meaningful. This distinction is important because the absence of multiplicative interaction does not rule out a clinically relevant high-risk subgroup that may benefit from integrated assessment of physical function and social health.

The metabolomic analyses provide exploratory evidence on potential biological pathways linking frailty, social disconnection, and PAD. The exposure-specific MRSs constructed in the training set remained associated with incident PAD in the validation set. However, their time-dependent AUCs were modest, indicating that these scores should be regarded as biological signatures rather than clinical prediction tools. These findings are consistent with prior NMR-based metabolomic studies showing that lipoprotein and metabolite profiles are associated with future PAD risk (39). Several selected biomarkers were related to systemic inflammation, lipoprotein lipid composition, and energy metabolism, including glycoprotein acetyls, 3-hydroxybutyrate, and lipid measures across lipoprotein subclasses. The repeated selection of glycoprotein acetyls is notable because this NMR-derived marker reflects systemic low-grade inflammation and has been linked to cardiometabolic and cardiovascular risk (40). Altered lipoprotein composition may reflect atherogenic lipid remodeling, whereas ketone-body related signals may indicate shifts in energy substrate utilization in metabolically vulnerable individuals. Recent proteomic evidence from UK Biobank also supports a biological connection between social disconnection and adverse health outcomes, showing that social isolation and loneliness are associated with plasma protein signatures involving inflammation, antiviral responses, and complement pathways (41). Together, these findings suggest that inflammatory, lipid-related, and immunometabolic dysregulation may represent shared biological pathways linking physical frailty, social disconnection, and atherosclerotic PAD risk.

In the validation set, the frailty-, loneliness-, and social isolation-related MRSs statistically accounted for 16.6, 20.4, and 22.6% of the corresponding associations with incident PAD. These findings suggest that the exposures and PAD share identifiable metabolomic patterns. However, these analyses represent exploratory statistical decomposition rather than established causal mediation. Because the exposures and metabolomic biomarkers were assessed at or near baseline, their temporal ordering could not be determined. Residual confounding and reverse causation also remain possible. The MRSs should therefore be regarded as biological signatures associated with both exposure status and PAD risk rather than causal mediators. The exploratory XGBoost analyses complemented the regression findings by visualizing feature-attribution patterns within the prespecified non-demographic feature set. Smoking status and eGFR showed the largest SHAP contributions in both models. Frailty score and the frailty-related MRS contributed more to the fitted model output than loneliness, social isolation, and their corresponding MRSs. Main SHAP contributions generally exceeded interaction contributions, although interaction patterns involving smoking status, eGFR, HbA1c, frailty, and the MRSs were observed. SHAP attribution depends on the included variables, their distributions, and correlations and should not be interpreted as an etiologic effect estimate or evidence of improved prediction. Accordingly, the lower SHAP contributions of loneliness and social isolation do not contradict their associations with PAD in the Cox models.

## 5 Strengths and limitations

This study has several strengths, including its prospective design, large sample of individuals with T2DM, long follow-up, and linkage to hospital inpatient and death registry records for PAD ascertainment. The analyses incorporated established measures of frailty and social disconnection, adjusted for a broad range of clinical, lifestyle, medication-related, and genetic factors, and were supported by subgroup and sensitivity analyses. In addition, the integration of NMR metabolomics, mediation analysis, and interpretable machine learning provided complementary evidence on potential metabolic signatures and predictive patterns.

Several limitations should be acknowledged. The observational design precludes causal inference, and residual confounding remains possible. Frailty was assessed using an adapted Fried phenotype based on available UK Biobank variables. Self-reported weight loss did not capture its intentionality or magnitude, and the grip-strength cutoffs, developed mainly in older adults, may be less accurate in younger participants, particularly women. Loneliness, social isolation, and frailty were measured only at baseline and may have changed during follow-up, potentially resulting in exposure misclassification. Fasting insulin was unavailable, precluding calculation of HOMA-IR and QUICKI. Although the TyG index was evaluated in sensitivity analyses, it remains an indirect surrogate of insulin resistance, particularly because blood samples were not collected under a uniform fasting protocol. The T2DM cohort was also heterogeneous with respect to disease duration, severity, treatment status, and endogenous insulin secretion. Although participants with possible type 1 diabetes were excluded, residual misclassification remained possible, and diabetes subclusters or phenotype-specific associations were not evaluated.

Of the 34,404 participants without prevalent PAD, 11,822 were excluded because of missing frailty or social-disconnection data. Although multiple-imputation analyses yielded similar results, the missing-at-random assumption cannot be verified, and residual selection bias may remain. The contemporaneous assessment of the exposures and metabolomic biomarkers precluded establishing temporal ordering. Moreover, the exploratory mediation estimates depended on the Weibull accelerated failure-time specification, and competing mortality was treated as censoring rather than modeled separately. These analyses should therefore be interpreted as statistical decomposition rather than causal mediation. The MRSs were evaluated in an internal holdout sample but lacked external validation. The exploratory XGBoost analyses used a binary 10-year PAD outcome, excluded participants censored before 10 years, omitted demographic characteristics a priori, and were not validated as clinical prediction models. SHAP values reflect attribution within the fitted models rather than causal effects, incremental predictive value, or clinical utility. PAD ascertainment through hospital and death registry records may have missed mild or asymptomatic cases. Finally, the predominantly White, middle-aged to older UK Biobank population may limit the generalizability of our findings to younger adults, Asian and other ethnically diverse populations, and low-income settings. The healthy-volunteer profile of UK Biobank and the availability of metabolomic and genetic data only in subsets may further restrict generalizability.

## 6 Conclusion

In conclusion, frailty, loneliness, and social isolation remained associated with incident PAD after mutual adjustment among individuals with T2DM, with the highest risk observed among participants with both frailty and social disconnection. In exploratory mediation analyses, exposure-specific metabolomic signatures statistically accounted for part of these associations. These findings suggest that physical vulnerability and social disconnection may represent complementary vascular risk domains in T2DM, while the metabolomic findings require external validation and should not be interpreted causally.

## Data Availability

The original contributions presented in the study are included in the article/Supplementary material, further inquiries can be directed to the corresponding authors.
