# Supplementary material for: Frailty, social disconnection, metabolomic signatures, and incident peripheral artery disease in type 2 diabetes: a prospective cohort study
Source: Front Nutr. 2026 Aug 21;13:1909900. doi: 10.3389/fnut.2026.1909900 (PMC13541745; doi:10.3389/fnut.2026.1909900)
Supplement: Supplementary file 1 [file Supplementary_file_1.docx]

**Frailty, Social Disconnection, Metabolomic Signatures, and Incident Peripheral Artery Disease in Type 2 Diabetes: A Prospective Cohort Study**

**Table S1.** Frailty phenotype and criteria for scoring

| **Individual components** | **Field**  **IDs** | **Field description** | **Coding criteria** |
| --- | --- | --- | --- |
| Weight Loss | 2306 | Compared with one year ago, has your weight changed | 1="Yes - lost weight"  0="No - weigh about the same" or "Yes - gained weight"  NA="Do not know" or "Prefer not to answer" |
| Slow gait speed | 924 | How would you describe your usual walking pace | 1="Slow pace"  0="Steady average pace" or "Brisk pace" or "None of the above"  NA="Do not know" or "Prefer not to answer" |
| Exhaustion | 2080 | Over the past two weeks, how often have you felt tired or had  little energy | 1="More than half the days" or "Nearly every day"  0="Not at all" or "Several days"  NA="Do not know" or "Prefer not to answer" |
| Low physical activity | 6164 | Types of physical activity in last 4 weeks | 1=none or light activity with a frequency of once per week or less |
|  | 2624 | Frequency of heavy DIY in last 4 weeks | 0=medium or heavy activity, or light activity more than once per week |
|  | 1011 | Frequency of light DIY in last 4 weeks |  |
|  | 3637 | Frequency of other exercises in last 4 weeks |  |
|  | 943 | Frequency of stair climbing in last 4 weeks |  |
|  | 991 | Frequency of strenuous sports in last 4 weeks |  |
|  | 971 | Frequency of walking for pleasure in last 4 weeks |  |
| Low grip strength | 31 | sex | The higher of the two measured grip strength expressed in |
|  | 21001  46  47 | Body mass index (BMI)  Hand grip strength (left)  Hand grip strength (right) | kg by sex- and BMI adjusted cut-off points.  Male  If BMI ≤24.0 kg/m2 & grip strength ≤29 kg  If BMI 24.1 to 26.0 kg/m2 & grip strength ≤30 kg  If BMI 26.1 to 28.0 kg/m2 & grip strength ≤30 kg If BMI >28.0 kg/m2 & grip strength ≤32 kg Female  If BMI ≤23.0 kg/m2 & grip strength ≤17 kg  If BMI 23.1 to 26.0 kg/m2 & grip strength ≤17.3 kg  If BMI 26.1 to 29.0 kg/m2 & grip strength ≤18 kg  If BMI >29.0 kg/m2 & grip strength ≤21 kg  If data on BMI or grip strength is not available = missing data. |

**Note:** Frailty phenotype was assessed using five components: weight loss, slow gait speed, exhaustion, low physical activity, and low grip strength. Each component was coded as 1 if the criterion was met and 0 if not met. Responses of “Do not know” or “Prefer not to answer” were coded as missing. Low physical activity was defined based on self-reported activity types and frequencies in the previous 4 weeks. Low grip strength was determined using the higher value of left- and right-hand grip strength according to sex- and body mass index-specific cut-off points; participants with missing body mass index or grip strength data were coded as missing for this component. BMI, body mass index; DIY, do-it-yourself; NA, not available.

**Table S2. ICD-10 diagnosis and OPCS-4 procedure codes used to ascertain peripheral artery disease**

| **Coding system** | **Application** | **Codes** |
| --- | --- | --- |
| ICD-10 | PAD diagnosis records before or at baseline defined prevalent PAD; the first qualifying record after baseline defined incident PAD. | I70.0; I70.00; I70.01; I70.2; I70.20; I70.21; I70.8; I70.80; I70.9; I70.90; I73.8; I73.9 |
| OPCS-4 | PAD-related procedures before or at baseline defined prevalent PAD; the first qualifying procedure after baseline defined incident PAD. | X09.3; X09.4; X09.5; L21.6; L51.3; L51.6; L51.8; L52.1; L52.2; L54.1; L54.4; L54.8; L59.1; L59.2; L59.3; L59.4; L59.5; L59.6; L59.7; L59.8; L60.1; L60.2; L63.1; L63.5; L63.9; L66.7 |

**Note:** Codes are presented using conventional punctuation, although the corresponding UK Biobank source fields may store them without decimal points. ICD-10, International Classification of Diseases, Tenth Revision; OPCS-4, Office of Population Censuses and Surveys Classification of Interventions and Procedures, version 4; PAD, peripheral artery disease.

**Table S3.** Assessment of Healthy Diet Score in the UK Biobank

| **Diet** | **UK Biobank field ID** | **Description** | **Healthy diet score** |
| --- | --- | --- | --- |
| Vegetable | 1289 | Cooked vegetable intake | 1 for ≥4 tablespoons/day |
|  | 1299 | Salad/raw vegetable intake | 0 for <4 tablespoons/day |
| Fruit | 1309 | Fresh fruit intake | 1 for ≥3 pieces/day |
|  | 1319 | Dried fruit intake | 0 for <3 pieces/day |
| Fish | 1329 | Oily fish intake | 1 for ≥2 times/week |
|  | 1339 | Non-oily fish intake | 0 for <2 times/week |
| Processed meat | 1349 | Processed meat intake | 1 for <2 times/week 0 for ≥2 times/week |
| Unprocessed red meat | 1369 | Beef intake | 1 for <2 times/week |
|  | 1379 | Lamb/mutton intake | 0 for ≥2 times/week |
|  | 1389 | Pork intake | 0 for ≥2 times/week |

**Note:** The healthy diet score was derived from UK Biobank dietary questionnaire items. Component criteria are shown above; scores were summed across components.

**Table S4. Missingness of individual frailty phenotype components**

| **Frailty component** | **UK Biobank field(s)** | **Missing, %** | **Principal source of missingness** |
| --- | --- | --- | --- |
| Low physical activity | 6164, 2624, 1011, 3637, 943, 991, and 971 | 15.8 | Information required to derive the component was unavailable in one or more contributing activity-frequency fields. |
| Exhaustion | 2080 | 7.4 | Response unavailable, including “Do not know” or “Prefer not to answer.” |
| Self-reported weight loss | 2306 | 3.1 | Response unavailable, including “Do not know” or “Prefer not to answer.” |
| Slow walking pace | 924 | 2.6 | Response unavailable, including “Do not know” or “Prefer not to answer.” |
| Low grip strength | 31, 21001, 46, and 47 | 3.3 | Grip-strength or body mass index data required to apply the sex- and body mass index-specific thresholds were unavailable. |

**Note.** Participant-specific reasons for missing measurements were not uniformly available in UK Biobank; therefore, the table reports the operational source of missingness. Missingness could overlap across components.

**Table S5. Baseline characteristics of participants with metabolomic data in the training and validation sets**

| **Characteristic** | **Overall (n = 21,986)** | **Training set (n = 15,390)** | **Validation set (n = 6,596)** | **P value** | **SMD** |
| --- | --- | --- | --- | --- | --- |
| Age, years | 59.288±7.213 | 59.315±7.205 | 59.227±7.232 | 0.407 | 0.012 |
| **Sex, n (%)** |  |  |  | 0.496 | 0.010 |
| Female | 13,603 (61.87) | 9,499 (61.72) | 4,104 (62.22) |  |  |
| Male | 8,383 (38.13) | 5,891 (38.28) | 2,492 (37.78) |  |  |
| **Ethnicity, n (%)** |  |  |  | 0.630 | 0.007 |
| Other | 2,083 (9.47) | 1,448 (9.41) | 635 (9.63) |  |  |
| White | 19,903 (90.53) | 13,942 (90.59) | 5,961 (90.37) |  |  |
| **Educational attainment, n (%)** |  |  |  | 0.679 | 0.006 |
| Other | 15,667 (71.26) | 10,980 (71.35) | 4,687 (71.06) |  |  |
| College/university or above | 6,319 (28.74) | 4,410 (28.65) | 1,909 (28.94) |  |  |
| Townsend Deprivation Index | -1.68 (-3.4-1.35) | -1.72 (-3.43-1.32) | -1.6 (-3.322-1.433) | 0.023 | 0.033 |
| **Smoking status, n (%)** |  |  |  | 0.358 | 0.021 |
| Never | 10,230 (46.53) | 7,176 (46.63) | 3,054 (46.30) |  |  |
| Previous | 9,453 (43.00) | 6,578 (42.74) | 2,875 (43.59) |  |  |
| Current | 2,303 (10.47) | 1,636 (10.63) | 667 (10.11) |  |  |
| **Healthy diet, n (%)** |  |  |  | 0.055 | 0.028 |
| No | 10,991 (49.99) | 7,628 (49.56) | 3,363 (50.99) |  |  |
| Yes | 10,995 (50.01) | 7,762 (50.44) | 3,233 (49.01) |  |  |
| **Loneliness score, n (%)** |  |  |  | 0.210 | 0.026 |
| 0 | 13,714 (62.38) | 9,656 (62.74) | 4,058 (61.52) |  |  |
| 1 | 6,340 (28.84) | 4,387 (28.51) | 1,953 (29.61) |  |  |
| 2 | 1,932 (8.79) | 1,347 (8.75) | 585 (8.87) |  |  |
| **Social isolation score, n (%)** |  |  |  | 0.140 | 0.034 |
| 0 | 10,323 (46.95) | 7,297 (47.41) | 3,026 (45.88) |  |  |
| 1 | 8,939 (40.66) | 6,223 (40.44) | 2,716 (41.18) |  |  |
| 2 | 2,465 (11.21) | 1,695 (11.01) | 770 (11.67) |  |  |
| 3 | 259 (1.18) | 175 (1.14) | 84 (1.27) |  |  |
| **Frailty phenotype score, n (%)** |  |  |  | 0.074 | 0.047 |
| 0 | 7,420 (33.75) | 5,282 (34.32) | 2,138 (32.41) |  |  |
| 1 | 7,948 (36.15) | 5,534 (35.96) | 2,414 (36.60) |  |  |
| 2 | 4,139 (18.83) | 2,846 (18.49) | 1,293 (19.60) |  |  |
| 3 | 1,778 (8.09) | 1,247 (8.10) | 531 (8.05) |  |  |
| 4 | 599 (2.72) | 407 (2.64) | 192 (2.91) |  |  |
| 5 | 102 (0.46) | 74 (0.48) | 28 (0.42) |  |  |
| Body mass index, kg/m2 | 30.024 (26.748-34.02) | 29.924 (26.649-33.908) | 30.247 (26.96-34.213) | 0.001 | 0.049 |
| Mean arterial pressure, mmHg | 101.85±11.231 | 101.856±11.173 | 101.835±11.365 | 0.899 | 0.002 |
| LDL-C, mmol/L | 2.783 (2.28-3.459) | 2.787 (2.291-3.464) | 2.772 (2.263-3.441) | 0.154 | 0.021 |
| HbA1c, % | 6.588 (5.892-7.366) | 6.588 (5.892-7.375) | 6.569 (5.883-7.347) | 0.302 | 0.018 |
| eGFR, mL/min/1.73 m2 | 88.191 (77.303-98.367) | 88.306 (77.434-98.454) | 88.028 (76.964-98.179) | 0.126 | 0.022 |
| **Lipid-lowering medication, n (%)** |  |  |  | 0.101 | 0.024 |
| No | 8,462 (38.49) | 5,978 (38.84) | 2,484 (37.66) |  |  |
| Yes | 13,524 (61.51) | 9,412 (61.16) | 4,112 (62.34) |  |  |
| **Antihypertensive medication, n (%)** |  |  |  | 0.301 | 0.015 |
| No | 9,958 (45.29) | 7,006 (45.52) | 2,952 (44.75) |  |  |
| Yes | 12,028 (54.71) | 8,384 (54.48) | 3,644 (55.25) |  |  |
| **Insulin use, n (%)** |  |  |  | 0.373 | 0.013 |
| No | 18,637 (84.77) | 13,068 (84.91) | 5,569 (84.43) |  |  |
| Yes | 3,349 (15.23) | 2,322 (15.09) | 1,027 (15.57) |  |  |
| **Aspirin use, n (%)** |  |  |  | 0.513 | 0.010 |
| No | 12,975 (59.01) | 9,060 (58.87) | 3,915 (59.35) |  |  |
| Yes | 9,011 (40.99) | 6,330 (41.13) | 2,681 (40.65) |  |  |
| **Incident PAD, n (%)** | 1,830 (8.32) | 1,281 (8.32) | 549 (8.32) | 1.000 | 0.000 |

**Note.** Continuous variables are presented as mean ± standard deviation or median (interquartile range), and categorical variables as n (%). eGFR, estimated glomerular filtration rate; HbA1c, glycated hemoglobin; LDL-C, low-density lipoprotein cholesterol; PAD, peripheral artery disease; SMD, standardized mean difference.

**Table S6. Metabolites and LASSO-Cox coefficients used to construct the exposure-specific metabolomic risk scores in the training set**

| **Metabolomic risk score** | **Selected metabolite** | **Coefficient** |
| --- | --- | --- |
| **Frailty-related MRS** | Glycoprotein Acetyls | 0.1064 |
|  | Phospholipids to Total Lipids in Very Small VLDL Percentage | 0.0799 |
|  | Polyunsaturated Fatty Acids | -0.0772 |
|  | Cholesteryl Esters to Total Lipids in IDL Percentage | -0.0764 |
|  | Degree of Unsaturation | -0.0551 |
|  | 3-Hydroxybutyrate | 0.0379 |
|  | Total Lipids in Medium HDL | -0.0322 |
|  | Albumin | -0.0281 |
|  | Total Concentration of Lipoprotein Particles | -0.0179 |
|  | Cholesteryl Esters in Small HDL | -0.0114 |
|  | Acetate | 0.0072 |
| **Loneliness-related MRS** | Phospholipids to Total Lipids in Very Small VLDL Percentage | 0.1047 |
|  | Cholesteryl Esters to Total Lipids in IDL Percentage | -0.0951 |
|  | Glycoprotein Acetyls | 0.0375 |
|  | Docosahexaenoic Acid | -0.0372 |
|  | Degree of Unsaturation | -0.0343 |
|  | Albumin | -0.0183 |
| **Social isolation-related MRS** | Total Concentration of Lipoprotein Particles | -0.1015 |
|  | Glycoprotein Acetyls | 0.0865 |
|  | Cholesteryl Esters to Total Lipids in IDL Percentage | -0.0808 |
|  | Phospholipids to Total Lipids in Very Small VLDL Percentage | 0.0706 |
|  | 3-Hydroxybutyrate | 0.0407 |
|  | Degree of Unsaturation | -0.0395 |
|  | Omega-3 Fatty Acids | -0.0381 |
|  | Albumin | -0.0279 |
|  | Linoleic Acid to Total Fatty Acids Percentage | -0.0177 |

**Note.** Only metabolites with nonzero coefficients at the one-standard-error lambda are shown. Each MRS was calculated as the weighted sum of the selected standardized metabolites. HDL, high-density lipoprotein; IDL, intermediate-density lipoprotein; LASSO, least absolute shrinkage and selection operator; MRS, metabolomic risk score; VLDL, very-low-density lipoprotein.

**Table S7. Associations of exposure-specific metabolomic risk scores with incident peripheral artery disease in the training set**

| **Metabolomic risk score** | **Category** | **PAD cases/total, n (%)** | **Unadjusted HR (95% CI)** | **P value** | **Adjusted HR (95% CI)** | **P value** |
| --- | --- | --- | --- | --- | --- | --- |
| **Frailty-related MRS** | T1 | 294/5,130 (5.73) | Reference |  | Reference |  |
|  | T2 | 409/5,130 (7.97) | 1.44 (1.24-1.67) | <0.001 | 1.20 (1.03-1.40) | 0.019 |
|  | T3 | 578/5,130 (11.27) | 2.15 (1.87-2.48) | <0.001 | 1.56 (1.34-1.82) | <0.001 |
|  | Per 1-SD increase | 1,281/15,390 (8.32) | 1.39 (1.31-1.46) | <0.001 | 1.22 (1.15-1.29) | <0.001 |
| **Loneliness-related MRS** | T1 | 309/5,130 (6.02) | Reference |  | Reference |  |
|  | T2 | 426/5,130 (8.30) | 1.43 (1.24-1.66) | <0.001 | 1.20 (1.04-1.40) | 0.016 |
|  | T3 | 546/5,130 (10.64) | 1.92 (1.67-2.21) | <0.001 | 1.50 (1.29-1.74) | <0.001 |
|  | Per 1-SD increase | 1,281/15,390 (8.32) | 1.33 (1.26-1.39) | <0.001 | 1.22 (1.15-1.30) | <0.001 |
| **Social isolation-related MRS** | T1 | 294/5,130 (5.73) | Reference |  | Reference |  |
|  | T2 | 417/5,130 (8.13) | 1.47 (1.27-1.70) | <0.001 | 1.22 (1.05-1.42) | 0.011 |
|  | T3 | 570/5,130 (11.11) | 2.12 (1.84-2.44) | <0.001 | 1.52 (1.30-1.77) | <0.001 |
|  | Per 1-SD increase | 1,281/15,390 (8.32) | 1.39 (1.32-1.46) | <0.001 | 1.21 (1.14-1.29) | <0.001 |

**Note.** The adjusted model included age, sex, ethnicity, Townsend Deprivation Index, educational attainment, smoking status, alcohol consumption, body mass index, mean arterial pressure, lipid-lowering medication, insulin use, aspirin use, antihypertensive medication, low-density lipoprotein cholesterol, glycated hemoglobin, estimated glomerular filtration rate, and dietary pattern. CI, confidence interval; HR, hazard ratio; MRS, metabolomic risk score; PAD, peripheral artery disease; SD, standard deviation.

**Table S8. Associations of exposure-specific metabolomic risk scores with incident peripheral artery disease in the validation set**

| **Metabolomic risk score** | **Category** | **PAD cases/total, n (%)** | **Unadjusted HR (95% CI)** | **P value** | **Adjusted HR (95% CI)** | **P value** |
| --- | --- | --- | --- | --- | --- | --- |
| **Frailty-related MRS** | T1 | 118/2,198 (5.37) | Reference |  | Reference |  |
|  | T2 | 188/2,198 (8.55) | 1.65 (1.31-2.08) | <0.001 | 1.22 (0.96-1.55) | 0.100 |
|  | T3 | 243/2,200 (11.05) | 2.23 (1.79-2.78) | <0.001 | 1.46 (1.15-1.85) | 0.002 |
|  | Per 1-SD increase | 549/6,596 (8.32) | 1.42 (1.31-1.53) | <0.001 | 1.22 (1.11-1.34) | <0.001 |
| **Loneliness-related MRS** | T1 | 119/2,198 (5.41) | Reference |  | Reference |  |
|  | T2 | 180/2,198 (8.19) | 1.58 (1.25-1.99) | <0.001 | 1.21 (0.96-1.54) | 0.111 |
|  | T3 | 250/2,200 (11.36) | 2.26 (1.82-2.81) | <0.001 | 1.61 (1.27-2.04) | <0.001 |
|  | Per 1-SD increase | 549/6,596 (8.32) | 1.35 (1.25-1.46) | <0.001 | 1.23 (1.12-1.34) | <0.001 |
| **Social isolation-related MRS** | T1 | 116/2,198 (5.28) | Reference |  | Reference |  |
|  | T2 | 188/2,198 (8.55) | 1.68 (1.33-2.12) | <0.001 | 1.23 (0.96-1.56) | 0.096 |
|  | T3 | 245/2,200 (11.14) | 2.30 (1.84-2.86) | <0.001 | 1.48 (1.17-1.88) | 0.001 |
|  | Per 1-SD increase | 549/6,596 (8.32) | 1.42 (1.31-1.54) | <0.001 | 1.22 (1.11-1.33) | <0.001 |

**Note.** MRSs in the validation set were calculated using metabolites and coefficients selected in the training set. The adjusted model included the same covariates as Table S7. CI, confidence interval; HR, hazard ratio; MRS, metabolomic risk score; PAD, peripheral artery disease; SD, standard deviation.

**Table S9. Time-dependent areas under the receiver operating characteristic curves for exposure-specific metabolomic risk scores in the training and validation sets**

| **Dataset** | **Metabolomic risk score** | **5-year AUC (95% CI)** | **10-year AUC (95% CI)** | **15-year AUC (95% CI)** |
| --- | --- | --- | --- | --- |
| **Training set** | Frailty-related MRS | 0.620 (0.596-0.649) | 0.616 (0.600-0.635) | 0.607 (0.592-0.621) |
|  | Loneliness-related MRS | 0.605 (0.571-0.631) | 0.597 (0.578-0.617) | 0.596 (0.582-0.611) |
|  | Social isolation-related MRS | 0.620 (0.586-0.651) | 0.617 (0.599-0.642) | 0.608 (0.590-0.623) |
| **Validation set** | Frailty-related MRS | 0.636 (0.592-0.683) | 0.608 (0.579-0.635) | 0.613 (0.590-0.640) |
|  | Loneliness-related MRS | 0.624 (0.587-0.670) | 0.602 (0.574-0.631) | 0.611 (0.587-0.629) |
|  | Social isolation-related MRS | 0.637 (0.596-0.685) | 0.609 (0.583-0.633) | 0.614 (0.593-0.642) |

**Note.** AUC, area under the time-dependent receiver operating characteristic curve; CI, confidence interval; MRS, metabolomic risk score.

**Table S10. Exploratory mediation analysis of the associations of frailty, loneliness, and social isolation with incident peripheral artery disease through corresponding metabolomic risk scores in the holdout validation set**

| Exposure | Mediator | Effect | Estimate (95% CI) | P value |
| --- | --- | --- | --- | --- |
| Frailty phenotype score | Frailty-related MRS | Indirect effect (control) | −6.11 (−8.32 to −4.59) | <0.001 |
|  |  | Indirect effect (treated) | −5.07 (−7.02 to −3.79) | <0.001 |
|  |  | Indirect effect (average) | −5.59 (−7.67 to −4.19) | <0.001 |
|  |  | Direct effect (control) | −28.84 (−37.64 to −22.07) | <0.001 |
|  |  | Direct effect (treated) | −27.79 (−36.27 to −21.29) | <0.001 |
|  |  | Direct effect (average) | −28.31 (−36.96 to −21.68) | <0.001 |
|  |  | Total effect | −33.90 (−43.57 to −26.67) | <0.001 |
|  |  | Proportion accounted for (control) | 18.1% (14.0%–23.3%) | <0.001 |
|  |  | Proportion accounted for (treated) | 15.0% (11.4%–19.6%) | <0.001 |
|  |  | Proportion accounted for (average) | 16.6% (12.7%–21.4%) | <0.001 |
| Loneliness | Loneliness-related MRS | Indirect effect (control) | −3.45 (−4.82 to −2.51) | <0.001 |
|  |  | Indirect effect (treated) | −3.12 (−4.26 to −2.23) | <0.001 |
|  |  | Indirect effect (average) | −3.29 (−4.63 to −2.38) | <0.001 |
|  |  | Direct effect (control) | −13.25 (−23.52 to −4.28) | <0.001 |
|  |  | Direct effect (treated) | −12.92 (−23.01 to −4.17) | <0.001 |
|  |  | Direct effect (average) | −13.09 (−23.26 to −4.22) | <0.001 |
|  |  | Total effect | −16.38 (−26.27 to −7.58) | <0.001 |
|  |  | Proportion accounted for (control) | 21.3% (13.0%–46.1%) | <0.001 |
|  |  | Proportion accounted for (treated) | 19.4% (11.2%–44.4%) | <0.001 |
|  |  | Proportion accounted for (average) | 20.4% (12.1%–45.2%) | <0.001 |
| Social isolation | Social isolation-related MRS | Indirect effect (control) | −4.34 (−5.90 to −3.25) | <0.001 |
|  |  | Indirect effect (treated) | −3.91 (−5.21 to −2.89) | <0.001 |
|  |  | Indirect effect (average) | −4.12 (−5.65 to −3.07) | <0.001 |
|  |  | Direct effect (control) | −14.25 (−24.17 to −5.44) | <0.001 |
|  |  | Direct effect (treated) | −13.82 (−23.54 to −5.27) | <0.001 |
|  |  | Direct effect (average) | −14.03 (−23.85 to −5.35) | <0.001 |
|  |  | Total effect | −18.16 (−27.85 to −9.51) | <0.001 |
|  |  | Proportion accounted for (control) | 23.8% (15.8%–44.7%) | <0.001 |
|  |  | Proportion accounted for (treated) | 21.5% (13.5%–42.9%) | <0.001 |
|  |  | Proportion accounted for (average) | 22.6% (14.7%–43.8%) | <0.001 |

*Note.* Estimates and 95% CIs were obtained from 1,000 quasi-Bayesian Monte Carlo simulations using the R package mediation. The outcome model was a Weibull accelerated failure-time model fitted using survreg. Control and treated denote the reference and exposed conditions, respectively; average is their mean. Non-proportion estimates are expressed on the survival-time scale used in the analysis; negative values indicate a shorter time to incident PAD. Proportions are expressed as percentages. Because the exposures and MRSs were assessed at or near baseline, the results represent exploratory statistical decomposition rather than causal mediation. CI, confidence interval; MRS, metabolomic risk score; PAD, peripheral artery disease.

**Table S11. Exploratory mediation estimates and E-value sensitivity analysis in the holdout validation set**

| Exposure | Average ACME (95% CI) | Adjusted HR (95% CI) per 1-SD increase in MRS | E-value for point estimate | E-value for CI limit |
| --- | --- | --- | --- | --- |
| Frailty phenotype score | −5.59 (−7.67 to −4.19) | 1.22 (1.11–1.34) | 1.74 | 1.46 |
| Loneliness | −3.29 (−4.63 to −2.38) | 1.23 (1.12–1.34) | 1.76 | 1.49 |
| Social isolation | −4.12 (−5.65 to −3.07) | 1.22 (1.11–1.33) | 1.74 | 1.46 |

**Note.** The average causal mediation effect (ACME) and its 95% CI were obtained from the Weibull accelerated failure-time mediation analysis; negative values indicate a shorter time to incident PAD. E-values were calculated separately from the multivariable-adjusted hazard ratios per 1-standard deviation increase in the corresponding MRS and from the confidence interval limit closest to the null. E-values were not calculated from the ACME estimates. Because PAD cumulative incidence was below 15%, hazard ratios were treated as approximations of risk ratios. CI, confidence interval; HR, hazard ratio; MRS, metabolomic risk score; PAD, peripheral artery disease; SD, standard deviation.

**Table S12. Baseline characteristics of participants included in and excluded from the complete-case sample**

| **Characteristic** | **Overall (n=32,838)** | **Missing (%)** | **Excluded (n=9,606)** | **Included (n=23,232)** | **P value** | **SMD** |
| --- | --- | --- | --- | --- | --- | --- |
| Age, years | 59.556 ± 7.145 | 0 | 60.042 ± 7.009 | 59.355 ± 7.190 | <0.001 | 0.097 |
| HbA1c, % | 6.606 (5.920–7.384) | 6.9 | 6.633 (5.975–7.420) | 6.588 (5.902–7.375) | 0.983 | <0.001 |
| eGFR, mL/min/1.73 m² | 85.642 ± 17.269 | 6.0 | 83.532 ± 17.987 | 86.489 ± 16.898 | <0.001 | 0.169 |
| Body mass index, kg/m² | 31.044 ± 5.899 | 0.9 | 31.618 ± 6.115 | 30.814 ± 5.794 | <0.001 | 0.135 |
| Townsend Deprivation Index | −0.602 ± 3.377 | 0.2 | −0.119 ± 3.510 | −0.801 ± 3.300 | <0.001 | 0.200 |
| **Sex, n (%)** |  | 0 |  |  | <0.001 | 0.217 |
| Female | 19,321 (58.84) |  | 4,924 (51.26) | 14,397 (61.97) |  |  |
| Male | 13,517 (41.16) |  | 4,682 (48.74) | 8,835 (38.03) |  |  |
| **Insulin use, n (%)** |  | 0.5 |  |  | 0.003 | 0.036 |
| No | 27,430 (83.98) |  | 7,831 (83.03) | 19,599 (84.37) |  |  |
| Yes | 5,231 (16.02) |  | 1,601 (16.97) | 3,630 (15.63) |  |  |
| **Hypertension, n (%)** |  | 0 |  |  | <0.001 | 0.043 |
| No | 6,519 (19.85) |  | 1,790 (18.63) | 4,729 (20.36) |  |  |
| Yes | 26,319 (80.15) |  | 7,816 (81.37) | 18,503 (79.64) |  |  |
| **Smoking status, n (%)** |  | 0.6 |  |  | <0.001 | 0.077 |
| Never | 15,150 (46.40) |  | 4,515 (47.53) | 10,635 (45.94) |  |  |
| Previous | 13,823 (42.34) |  | 3,795 (39.95) | 10,028 (43.32) |  |  |
| Current | 3,678 (11.26) |  | 1,190 (12.53) | 2,488 (10.75) |  |  |
| **PAD status, n (%)** |  | 0 |  |  | <0.001 | 0.063 |
| No PAD | 29,106 (88.64) |  | 8,397 (87.41) | 20,709 (89.14) |  |  |
| Incident PAD | 2,719 (8.28) |  | 846 (8.81) | 1,873 (8.06) |  |  |
| Prevalent PAD at baseline | 1,013 (3.08) |  | 363 (3.78) | 650 (2.80) |  |  |

**Note.** Values are presented as mean ± SD, median (IQR), or n (%), as appropriate. This comparison was restricted to participants with complete social-disconnection data and was conducted before the exclusion of prevalent PAD. Among these participants, 9,606 were excluded because of missing frailty data and 23,232 had complete exposure data. P values were obtained using the appropriate parametric, nonparametric, or categorical test. eGFR, estimated glomerular filtration rate; HbA1c, glycated hemoglobin; IQR, interquartile range; PAD, peripheral artery disease; SD, standard deviation; SMD, standardized mean difference.

**Table S13. Incident peripheral artery disease risk by inclusion status**

| **Main-analysis status** | **PAD cases/total, n (%)** | **Adjusted HR (95% CI)** | **P value** |
| --- | --- | --- | --- |
| Excluded because of missing exposure data | 846/9,243 (9.15) | Reference |  |
| Included in the main analysis | 1,873/22,582 (8.29) | 0.93 (0.86–1.01) | 0.077 |

**Note.** The analysis was restricted to participants with complete social-disconnection data and without prevalent PAD at baseline. The model was adjusted for age, sex, HbA1c, eGFR, body mass index, Townsend Deprivation Index, insulin use, hypertension, and smoking status. CI, confidence interval; eGFR, estimated glomerular filtration rate; HbA1c, glycated hemoglobin; HR, hazard ratio; PAD, peripheral artery disease.

**Table S14. Mutually adjusted associations of frailty, loneliness, and social isolation with incident peripheral artery disease**

| **Exposure** | **Category** | **PAD cases/total, n (%)** | **HR (95% CI)** | **P value** |
| --- | --- | --- | --- | --- |
| Loneliness | No loneliness | 1,662/20,594 (8.07) | Reference | — |
|  | Loneliness | 211/1,988 (10.61) | 1.19 (1.03–1.38) | 0.019 |
| Social isolation | No social isolation | 1,572/19,780 (7.95) | Reference | — |
|  | Social isolation | 301/2,802 (10.74) | 1.24 (1.10–1.41) | 0.001 |
| Frailty phenotype | Non-frail | 501/7,573 (6.62) | Reference | — |
|  | Pre-frail | 1,021/12,431 (8.21) | 1.22 (1.10–1.36) | <0.001 |
|  | Frail | 351/2,578 (13.62) | 2.02 (1.75–2.34) | <0.001 |

**Note.** Frailty phenotype, loneliness, and social isolation were included simultaneously in the Cox model. The model was additionally adjusted for age, sex, ethnicity, Townsend Deprivation Index, educational attainment, smoking status, alcohol consumption, body mass index, mean arterial pressure, lipid-lowering medication, insulin use, aspirin use, antihypertensive medication, low-density lipoprotein cholesterol, glycated hemoglobin, estimated glomerular filtration rate, and dietary pattern. CI, confidence interval; HR, hazard ratio; PAD, peripheral artery disease.

**Table S15. Collinearity diagnostics for the mutually adjusted Cox model**

| **Variable** | **GVIF** | **df** | **Adjusted GVIF*** |
| --- | --- | --- | --- |
| Frailty phenotype | 1.245 | 2 | 1.022 |
| Loneliness | 1.121 | 1 | 1.029 |
| Social isolation | 1.146 | 1 | 1.023 |
| Age | 1.286 | 1 | 1.134 |
| Sex | 1.119 | 1 | 1.058 |
| Ethnicity | 1.083 | 1 | 1.041 |
| Townsend Deprivation Index | 1.154 | 1 | 1.074 |
| Educational attainment | 1.038 | 1 | 1.019 |
| Body mass index | 1.231 | 1 | 1.109 |
| Mean arterial pressure | 1.113 | 1 | 1.055 |
| Lipid-lowering medication | 1.460 | 1 | 1.208 |
| Antihypertensive medication | 1.277 | 1 | 1.130 |
| Insulin use | 1.081 | 1 | 1.040 |
| Aspirin use | 1.196 | 1 | 1.094 |
| Low-density lipoprotein cholesterol | 1.426 | 1 | 1.194 |
| Glycated hemoglobin | 1.008 | 1 | 1.004 |
| Estimated glomerular filtration rate | 1.262 | 1 | 1.123 |
| Healthy dietary pattern | 1.055 | 1 | 1.027 |
| Smoking status | 1.139 | 2 | 1.033 |

**Note.** *Adjusted GVIF = GVIF^(1/(2 × df)). All adjusted GVIF values were ≤1.21, indicating no substantial collinearity. df, degrees of freedom; GVIF, generalized variance inflation factor.

**Table S16. Associations of individual frailty components with incident PAD**

| **Exposure** | **Cases/Total n (%)** | **Unadjusted HR (95% CI)** | **P value** | **Multivariable-adjusted HR (95% CI)** | **P value** |
| --- | --- | --- | --- | --- | --- |
| Low grip strength | 573/5,542 (10.34) | 1.44 (1.31-1.59) | <0.001 | 1.27 (1.15-1.40) | <0.001 |
| Low physical activity | 584/6,110 (9.56) | 1.27 (1.15-1.40) | <0.001 | 1.19 (1.07-1.31) | 0.001 |
| Weight loss | 453/5,576 (8.12) | 0.98 (0.88-1.09) | 0.682 | 1.07 (0.96-1.19) | 0.202 |
| Exhaustion | 410/3,951 (10.38) | 1.37 (1.23-1.53) | <0.001 | 1.36 (1.21-1.52) | <0.001 |
| Slow gait speed | 615/4,085 (15.06) | 2.55 (2.32-2.81) | <0.001 | 2.02 (1.82-2.24) | <0.001 |

**Note:** The multivariable-adjusted model included age, sex, ethnicity, Townsend Deprivation Index, educational attainment, smoking status, alcohol consumption, body mass index, mean arterial pressure, lipid-lowering medication, insulin use, aspirin use, antihypertensive medication, low-density lipoprotein cholesterol, glycated hemoglobin, estimated glomerular filtration rate, and dietary pattern. CI, confidence interval; eGFR, estimated glomerular filtration rate; HbA1c, glycated hemoglobin; HR, hazard ratio; LDL-C, low-density lipoprotein cholesterol; PAD, peripheral artery disease.

**Table S17. Adjusted population attributable fractions for incident PAD**

| **Factor** | **Adjusted PAF, % (95% CI)** |
| --- | --- |
| Slow gait speed | 15.61 (12.73-18.51) |
| Low grip strength | 6.17 (3.21-8.87) |
| Exhaustion | 5.92 (3.71-8.20) |
| Low physical activity | 4.80 (1.81-7.35) |
| Social isolation | 3.14 (1.12-5.04) |
| Loneliness | 2.19 (0.65-3.71) |
| Weight loss | 1.77 (-1.06-4.63) |

**Abbreviations:** CI, confidence interval; PAD, peripheral artery disease; PAF, population attributable fraction. PAFs were estimated from multivariable-adjusted models and should be interpreted cautiously in this observational study.

**Table S18. Associations between frailty phenotype and social disconnection**

**A. Modified Poisson regression: frailty phenotype and prevalence of social disconnection**

| **Outcome** | **Frailty phenotype** | **Cases/Total, n (%)** | **PR (95% CI)** | **P value** |
| --- | --- | --- | --- | --- |
| Loneliness | Non-frailty | 415/7,573 (5.48) | Ref |  |
|  | Pre-frailty | 1,119/12,431 (9.00) | 1.51 (1.35, 1.69) | <0.001 |
|  | Frailty | 454/2,578 (17.61) | 2.60 (2.27, 2.98) | <0.001 |
| Social isolation | Non-frailty | 673/7,573 (8.89) | Ref |  |
|  | Pre-frailty | 1,596/12,431 (12.84) | 1.34 (1.23, 1.46) | <0.001 |
|  | Frailty | 533/2,578 (20.67) | 1.89 (1.69, 2.11) | <0.001 |

**B. Ordinal logistic regression: social disconnection and ordered frailty status**

| **Exposure** | **Reference** | **OR (95% CI)** | **P value** |
| --- | --- | --- | --- |
| Loneliness | No loneliness | 1.91 (1.74, 2.10) | <0.001 |
| Social isolation | No social isolation | 1.56 (1.44, 1.70) | <0.001 |

**Note:** Models were multivariable adjusted. PR, prevalence ratio; OR, odds ratio; CI, confidence interval.

**Table S19. Joint associations and interaction between frailty phenotype and social disconnection with incident PAD**

**A. Joint Cox regression analysis for frailty phenotype and loneliness**

| **Frailty phenotype** | **Loneliness** | **HR (95% CI)** | **P value** |
| --- | --- | --- | --- |
| Non-frailty | No | Ref |  |
| Non-frailty | Yes | 1.20 (0.84, 1.73) | 0.320 |
| Pre-frailty | No | 1.21 (1.08, 1.36) | 0.001 |
| Pre-frailty | Yes | 1.68 (1.37, 2.06) | <0.001 |
| Frailty | No | 1.94 (1.47, 2.56) | <0.001 |
| Frailty | Yes | 2.17 (1.86, 2.54) | <0.001 |

**B. Joint Cox regression analysis for frailty phenotype and social isolation**

| **Frailty phenotype** | **Social isolation** | **HR (95% CI)** | **P value** |
| --- | --- | --- | --- |
| Non-frailty | No | Ref |  |
| Non-frailty | Yes | 1.19 (0.89, 1.59) | 0.237 |
| Pre-frailty | No | 1.23 (1.09, 1.38) | 0.001 |
| Pre-frailty | Yes | 1.34 (1.11, 1.61) | 0.003 |
| Frailty | No | 1.88 (1.60, 2.21) | <0.001 |
| Frailty | Yes | 2.79 (2.21, 3.51) | <0.001 |

**C. Interaction analyses**

| **Interaction** | **Measure** | **Estimate (95% CI)** |
| --- | --- | --- |
| Frailty score x loneliness score | RERI | 0.050 (0.002, 0.098) |
|  | AP | 0.038 (0.000, 0.076) |
|  | Multiplicative interaction | 1.028 (0.974, 1.085) |
| Frailty score x social isolation score | RERI | 0.063 (0.019, 0.106) |
|  | AP | 0.048 (0.012, 0.084) |
|  | Multiplicative interaction | 1.046 (0.995, 1.100) |

**D. Incidence rates across joint categories**

| **Frailty phenotype** | **Social disconnection** | **Indicator status** | **IR (95% CI)** |
| --- | --- | --- | --- |
| Non-frailty | Loneliness | No | 4.40 (4.01, 4.82) |
| Non-frailty | Loneliness | Yes | 5.14 (3.50, 7.30) |
| Pre-frailty | Loneliness | No | 5.49 (5.14, 5.86) |
| Pre-frailty | Loneliness | Yes | 7.66 (6.36, 9.16) |
| Frailty | Loneliness | No | 9.77 (7.44, 12.60) |
| Frailty | Loneliness | Yes | 10.70 (9.47, 12.00) |
| Non-frailty | Social isolation | No | 4.36 (3.97, 4.79) |
| Non-frailty | Social isolation | Yes | 5.27 (3.93, 6.93) |
| Pre-frailty | Social isolation | No | 5.50 (5.14, 5.88) |
| Pre-frailty | Social isolation | Yes | 6.96 (5.91, 8.15) |
| Frailty | Social isolation | No | 9.48 (8.35, 10.70) |
| Frailty | Social isolation | Yes | 14.80 (12.00, 18.10) |

**Note:** Models were multivariable adjusted. HR, hazard ratio; IR, incidence rate; CI, confidence interval; RERI, relative excess risk due to interaction; AP, attributable proportion due to interaction; PAD, peripheral artery disease.

**Table S20. Subgroup analyses of the associations of loneliness, social isolation, and frailty phenotype with incident PAD**

| **Subgroup** | **Category** | **Exposure status** | **Participants, n (%)** | **HR (95% CI)** | **P value** | **P for interaction** |
| --- | --- | --- | --- | --- | --- | --- |
| **Loneliness** | | | | | | |
| Age | <60 years | Yes | 9,601 (42.6) | 1.19 (0.93, 1.52) | 0.175 | 0.976 |
| Age | ≥60 years | Yes | 12,950 (57.4) | 1.24 (1.04, 1.48) | 0.019 |  |
| Sex | Female | Yes | 13,913 (61.7) | 1.25 (1.06, 1.48) | 0.009 | 0.910 |
| Sex | Male | Yes | 8,638 (38.3) | 1.25 (0.93, 1.67) | 0.132 |  |
| Obesity status | BMI <30 kg/m² | Yes | 11,207 (49.7) | 1.25 (0.99, 1.58) | 0.061 | 0.899 |
| Obesity status | BMI ≥30 kg/m² | Yes | 11,344 (50.3) | 1.24 (1.03, 1.49) | 0.025 |  |
| eGFR | ≥60 mL/min/1.73 m² | Yes | 21,161 (93.8) | 1.22 (1.04, 1.42) | 0.016 | 0.319 |
| eGFR | <60 mL/min/1.73 m² | Yes | 1,390 (6.2) | 1.41 (0.98, 2.03) | 0.062 |  |
| TDI | < median | Yes | 11,299 (50.0) | 1.12 (0.86, 1.44) | 0.407 | 0.164 |
| TDI | ≥ median | Yes | 11,283 (50.0) | 1.35 (1.14, 1.62) | 0.001 |  |
| Educational attainment | Below college/university degree | Yes | 16,080 (71.3) | 1.20 (1.02, 1.40) | 0.027 | 0.345 |
| Educational attainment | College/university degree or above | Yes | 6,471 (28.7) | 1.49 (1.05, 2.12) | 0.026 |  |
| Hypertension | No | Yes | 4,666 (20.7) | 1.59 (1.06, 2.39) | 0.024 | 0.254 |
| Hypertension | Yes | Yes | 17,885 (79.3) | 1.21 (1.04, 1.42) | 0.015 |  |
| Insulin use | No | Yes | 19,095 (84.7) | 1.32 (1.11, 1.55) | 0.001 | 0.331 |
| Insulin use | Yes | Yes | 3,456 (15.3) | 1.06 (0.79, 1.43) | 0.700 |  |
| Aspirin use | No | Yes | 13,300 (59.0) | 1.22 (0.98, 1.52) | 0.075 | 0.939 |
| Aspirin use | Yes | Yes | 9,251 (41.0) | 1.27 (1.05, 1.54) | 0.015 |  |
| Smoking status | Never | Yes | 10,511 (46.6) | 1.46 (1.13, 1.89) | 0.004 | 0.190 |
| Smoking status | Previous | Yes | 9,681 (42.9) | 1.13 (0.91, 1.40) | 0.285 |  |
| Smoking status | Current | Yes | 2,359 (10.5) | 1.30 (0.97, 1.75) | 0.079 |  |
| Alcohol consumption | Never | Yes | 1,426 (6.3) | 1.03 (0.56, 1.91) | 0.917 | 0.753 |
| Alcohol consumption | Previous | Yes | 1,418 (6.3) | 1.12 (0.71, 1.77) | 0.628 |  |
| Alcohol consumption | Current | Yes | 19,693 (87.4) | 1.27 (1.09, 1.49) | 0.003 |  |
| **Social isolation** | | | | | | |
| Age | <60 years | Yes | 9,601 (42.6) | 1.30 (1.04, 1.63) | 0.020 | 0.491 |
| Age | ≥60 years | Yes | 12,950 (57.4) | 1.23 (1.06, 1.44) | 0.008 |  |
| Sex | Female | Yes | 13,913 (61.7) | 1.24 (1.07, 1.44) | 0.004 | 0.719 |
| Sex | Male | Yes | 8,638 (38.3) | 1.30 (1.01, 1.67) | 0.044 |  |
| Obesity status | BMI <30 kg/m² | Yes | 11,207 (49.7) | 1.23 (1.01, 1.50) | 0.041 | 0.911 |
| Obesity status | BMI ≥30 kg/m² | Yes | 11,344 (50.3) | 1.27 (1.08, 1.50) | 0.005 |  |
| eGFR | ≥60 mL/min/1.73 m² | Yes | 21,161 (93.8) | 1.22 (1.06, 1.40) | 0.006 | 0.638 |
| eGFR | <60 mL/min/1.73 m² | Yes | 1,390 (6.2) | 1.26 (0.92, 1.72) | 0.155 |  |
| TDI | < median | Yes | 11,299 (50.0) | 1.27 (1.02, 1.59) | 0.036 | 0.714 |
| TDI | ≥ median | Yes | 11,283 (50.0) | 1.31 (1.13, 1.53) | <0.001 |  |
| Educational attainment | Below college/university degree | Yes | 16,080 (71.3) | 1.23 (1.07, 1.42) | 0.004 | 0.933 |
| Educational attainment | College/university degree or above | Yes | 6,471 (28.7) | 1.33 (0.98, 1.79) | 0.067 |  |
| Hypertension | No | Yes | 4,666 (20.7) | 1.25 (0.84, 1.87) | 0.279 | 0.953 |
| Hypertension | Yes | Yes | 17,885 (79.3) | 1.25 (1.09, 1.42) | 0.001 |  |
| Insulin use | No | Yes | 19,095 (84.7) | 1.26 (1.09, 1.45) | 0.002 | 0.823 |
| Insulin use | Yes | Yes | 3,456 (15.3) | 1.25 (0.97, 1.63) | 0.089 |  |
| Aspirin use | No | Yes | 13,300 (59.0) | 1.33 (1.10, 1.60) | 0.003 | 0.282 |
| Aspirin use | Yes | Yes | 9,251 (41.0) | 1.20 (1.01, 1.43) | 0.043 |  |
| Smoking status | Never | Yes | 10,511 (46.6) | 1.43 (1.14, 1.79) | 0.002 | 0.391 |
| Smoking status | Previous | Yes | 9,681 (42.9) | 1.20 (1.00, 1.46) | 0.056 |  |
| Smoking status | Current | Yes | 2,359 (10.5) | 1.17 (0.90, 1.51) | 0.239 |  |
| Alcohol consumption | Never | Yes | 1,426 (6.3) | 1.32 (0.81, 2.14) | 0.268 | 0.661 |
| Alcohol consumption | Previous | Yes | 1,418 (6.3) | 1.47 (1.02, 2.12) | 0.037 |  |
| Alcohol consumption | Current | Yes | 19,693 (87.4) | 1.20 (1.04, 1.39) | 0.012 |  |
| **Frailty phenotype** | | | | | | |
| Age | <60 years | Pre-frailty | 9,601 (42.6) | 1.32 (1.06, 1.65) | 0.012 | 0.383 |
| Age | <60 years | Frailty | 9,601 (42.6) | 2.30 (1.75, 3.02) | <0.001 |  |
| Age | ≥60 years | Pre-frailty | 12,950 (57.4) | 1.19 (1.05, 1.35) | 0.006 |  |
| Age | ≥60 years | Frailty | 12,950 (57.4) | 1.95 (1.64, 2.31) | <0.001 |  |
| Sex | Female | Pre-frailty | 13,913 (61.7) | 1.27 (1.12, 1.43) | <0.001 | 0.143 |
| Sex | Female | Frailty | 13,913 (61.7) | 1.94 (1.64, 2.31) | <0.001 |  |
| Sex | Male | Pre-frailty | 8,638 (38.3) | 1.07 (0.85, 1.35) | 0.540 |  |
| Sex | Male | Frailty | 8,638 (38.3) | 2.13 (1.61, 2.81) | <0.001 |  |
| Obesity status | BMI <30 kg/m² | Pre-frailty | 11,207 (49.7) | 1.16 (1.00, 1.35) | 0.051 | 0.831 |
| Obesity status | BMI <30 kg/m² | Frailty | 11,207 (49.7) | 1.99 (1.57, 2.51) | <0.001 |  |
| Obesity status | BMI ≥30 kg/m² | Pre-frailty | 11,344 (50.3) | 1.25 (1.06, 1.46) | 0.007 |  |
| Obesity status | BMI ≥30 kg/m² | Frailty | 11,344 (50.3) | 2.01 (1.66, 2.43) | <0.001 |  |
| eGFR | ≥60 mL/min/1.73 m² | Pre-frailty | 21,161 (93.8) | 1.20 (1.07, 1.34) | 0.002 | 0.761 |
| eGFR | ≥60 mL/min/1.73 m² | Frailty | 21,161 (93.8) | 1.93 (1.64, 2.26) | <0.001 |  |
| eGFR | <60 mL/min/1.73 m² | Pre-frailty | 1,390 (6.2) | 1.37 (0.95, 1.99) | 0.096 |  |
| eGFR | <60 mL/min/1.73 m² | Frailty | 1,390 (6.2) | 2.05 (1.35, 3.10) | 0.001 |  |
| TDI | < median | Pre-frailty | 11,299 (50.0) | 1.25 (1.07, 1.45) | 0.004 | 0.828 |
| TDI | < median | Frailty | 11,299 (50.0) | 2.04 (1.62, 2.57) | <0.001 |  |
| TDI | ≥ median | Pre-frailty | 11,283 (50.0) | 1.21 (1.03, 1.41) | 0.018 |  |
| TDI | ≥ median | Frailty | 11,283 (50.0) | 2.05 (1.69, 2.48) | <0.001 |  |
| Educational attainment | Below college/university degree | Pre-frailty | 16,080 (71.3) | 1.25 (1.10, 1.41) | <0.001 | 0.300 |
| Educational attainment | Below college/university degree | Frailty | 16,080 (71.3) | 2.12 (1.80, 2.48) | <0.001 |  |
| Educational attainment | College/university degree or above | Pre-frailty | 6,471 (28.7) | 1.09 (0.86, 1.38) | 0.469 |  |
| Educational attainment | College/university degree or above | Frailty | 6,471 (28.7) | 1.48 (1.02, 2.16) | 0.041 |  |
| Hypertension | No | Pre-frailty | 4,666 (20.7) | 1.35 (0.98, 1.86) | 0.068 | 0.374 |
| Hypertension | No | Frailty | 4,666 (20.7) | 1.57 (0.93, 2.65) | 0.090 |  |
| Hypertension | Yes | Pre-frailty | 17,885 (79.3) | 1.21 (1.07, 1.35) | 0.002 |  |
| Hypertension | Yes | Frailty | 17,885 (79.3) | 2.04 (1.75, 2.37) | <0.001 |  |
| Insulin use | No | Pre-frailty | 19,095 (84.7) | 1.18 (1.04, 1.33) | 0.009 | 0.252 |
| Insulin use | No | Frailty | 19,095 (84.7) | 1.91 (1.61, 2.26) | <0.001 |  |
| Insulin use | Yes | Pre-frailty | 3,456 (15.3) | 1.39 (1.09, 1.78) | 0.007 |  |
| Insulin use | Yes | Frailty | 3,456 (15.3) | 2.29 (1.70, 3.07) | <0.001 |  |
| Aspirin use | No | Pre-frailty | 13,300 (59.0) | 1.18 (1.00, 1.38) | 0.047 | 0.928 |
| Aspirin use | No | Frailty | 13,300 (59.0) | 1.91 (1.54, 2.38) | <0.001 |  |
| Aspirin use | Yes | Pre-frailty | 9,251 (41.0) | 1.25 (1.08, 1.45) | 0.004 |  |
| Aspirin use | Yes | Frailty | 9,251 (41.0) | 2.06 (1.69, 2.50) | <0.001 |  |
| Smoking status | Never | Pre-frailty | 10,511 (46.6) | 1.20 (0.99, 1.46) | 0.070 | 0.769 |
| Smoking status | Never | Frailty | 10,511 (46.6) | 2.12 (1.63, 2.75) | <0.001 |  |
| Smoking status | Previous | Pre-frailty | 9,681 (42.9) | 1.24 (1.07, 1.44) | 0.005 |  |
| Smoking status | Previous | Frailty | 9,681 (42.9) | 1.95 (1.59, 2.40) | <0.001 |  |
| Smoking status | Current | Pre-frailty | 2,359 (10.5) | 1.18 (0.90, 1.56) | 0.235 |  |
| Smoking status | Current | Frailty | 2,359 (10.5) | 1.91 (1.37, 2.66) | <0.001 |  |
| Alcohol consumption | Never | Pre-frailty | 1,426 (6.3) | 1.35 (0.77, 2.35) | 0.291 | 0.954 |
| Alcohol consumption | Never | Frailty | 1,426 (6.3) | 2.38 (1.27, 4.45) | 0.007 |  |
| Alcohol consumption | Previous | Pre-frailty | 1,418 (6.3) | 1.16 (0.74, 1.83) | 0.520 |  |
| Alcohol consumption | Previous | Frailty | 1,418 (6.3) | 2.09 (1.26, 3.48) | 0.004 |  |
| Alcohol consumption | Current | Pre-frailty | 19,693 (87.4) | 1.21 (1.08, 1.36) | 0.001 |  |
| Alcohol consumption | Current | Frailty | 19,693 (87.4) | 1.95 (1.66, 2.29) | <0.001 |  |

**Note.** Hazard ratios were estimated using multivariable-adjusted Cox proportional hazards models. For loneliness and social isolation, participants without the corresponding exposure were used as the reference group; “Yes” indicates the presence of loneliness or social isolation. For frailty phenotype, non-frail participants were used as the reference group, and pre-frail and frail participants were compared with non-frail participants. P values for interaction were calculated by adding cross-product terms between each exposure and the subgroup variable. BMI, body mass index; CI, confidence interval; eGFR, estimated glomerular filtration rate; HR, hazard ratio; PAD, peripheral artery disease; TDI, Townsend Deprivation Index.

**Table S21. Sensitivity analyses of the associations of loneliness, social isolation, and frailty phenotype with incident PAD**

**A. Main associations**

| **Exposure** | **Category** | **Cases/Total n (%)** | **HR (95% CI)** | **P value** |
| --- | --- | --- | --- | --- |
| Loneliness scale | No loneliness | 1,619/20,102 (8.05) | Ref |  |
|  | Loneliness | 206/1,926 (10.70) | 1.26 (1.09, 1.46) | 0.002 |
|  | Per one-point increase | 1,825/22,028 (8.28) | 1.16 (1.08, 1.24) | <0.001 |
| Social isolation scale | No social isolation | 1,534/19,312 (7.94) | Ref |  |
|  | Social isolation | 291/2,716 (10.71) | 1.26 (1.11, 1.43) | <0.001 |
|  | Per one-point increase | 1,825/22,028 (8.28) | 1.14 (1.07, 1.22) | <0.001 |
| Frailty phenotype | Non-frailty | 494/7,428 (6.65) | Ref |  |
|  | Pre-frailty | 988/12,113 (8.16) | 1.20 (1.08, 1.34) | 0.001 |
|  | Frailty | 343/2,487 (13.79) | 2.01 (1.74, 2.33) | <0.001 |
|  | Per one-point increase | 1,825/22,028 (8.28) | 1.25 (1.20, 1.31) | <0.001 |

**B. Joint associations**

| **Joint exposure** | **Frailty phenotype** | **Social disconnection** | **HR (95% CI)** | **P value** |
| --- | --- | --- | --- | --- |
| Frailty phenotype and loneliness | Non-frailty | No loneliness | Ref |  |
|  | Non-frailty | Loneliness | 1.21 (0.84, 1.75) | 0.300 |
|  | Pre-frailty | No loneliness | 1.20 (1.06, 1.34) | 0.002 |
|  | Pre-frailty | Loneliness | 1.68 (1.37, 2.06) | <0.001 |
|  | Frailty | No loneliness | 1.97 (1.49, 2.60) | <0.001 |
|  | Frailty | Loneliness | 2.16 (1.84, 2.52) | <0.001 |
| Frailty phenotype and social isolation | Non-frailty | No social isolation | Ref |  |
|  | Non-frailty | Social isolation | 1.18 (0.88, 1.58) | 0.273 |
|  | Pre-frailty | No social isolation | 1.21 (1.08, 1.36) | 0.001 |
|  | Pre-frailty | Social isolation | 1.32 (1.09, 1.59) | 0.005 |
|  | Frailty | No social isolation | 1.86 (1.58, 2.19) | <0.001 |
|  | Frailty | Social isolation | 2.84 (2.25, 3.58) | <0.001 |

**C. Interaction analyses**

| **Interaction term** | **Measure** | **Estimate (95% CI)** |
| --- | --- | --- |
| Frailty score x loneliness score | RERI | 0.053 (0.005, 0.101) |
|  | AP | 0.040 (0.002, 0.079) |
|  | Multiplicative interaction | 1.035 (0.980, 1.092) |
| Frailty score x social isolation score | RERI | 0.068 (0.023, 0.112) |
|  | AP | 0.052 (0.016, 0.089) |
|  | Multiplicative interaction | 1.051 (0.998, 1.106) |

**Note:** Models were multivariable adjusted. HR, hazard ratio; CI, confidence interval; PAD, peripheral artery disease; RERI, relative excess risk due to interaction; AP, attributable proportion due to interaction. P values <0.001 are shown as <0.001.

**Table S22. Sensitivity analyses using Fine-Gray competing risk models for incident PAD**

**A. Main associations**

| **Exposure** | **Category** | **Cases/Total n (%)** | **sHR (95% CI)** | **P value** |
| --- | --- | --- | --- | --- |
| Loneliness | No Loneliness | 1,662/20,594 (8.07) | Ref |  |
| Loneliness | Loneliness | 211/1,988 (10.61) | 1.24 (1.07-1.43) | 0.005 |
| Loneliness | Per one point increase | 1,873/22,582 (8.29) | 1.14 (1.06-1.22) | <0.001 |
| Social isolation | No Social isolation | 1,572/19,780 (7.95) | Ref |  |
| Social isolation | Social isolation | 301/2,802 (10.74) | 1.19 (1.05-1.35) | 0.008 |
| Social isolation | Per one point increase | 1,873/22,582 (8.29) | 1.11 (1.04-1.18) | 0.001 |
| Frailty phenotype | Non-frailty | 501/7,573 (6.62) | Ref |  |
| Frailty phenotype | Pre-frailty | 1,021/12,431 (8.21) | 1.19 (1.06-1.32) | 0.002 |
| Frailty phenotype | Frailty | 351/2,578 (13.62) | 1.81 (1.54-2.12) | <0.001 |
| Frailty phenotype | Per one point increase | 1,873/22,582 (8.29) | 1.21 (1.16-1.26) | <0.001 |

**B. Joint associations**

| **Joint exposure** | **Frailty phenotype** | **Social disconnection** | **sHR (95% CI)** | **P value** |
| --- | --- | --- | --- | --- |
| Frailty phenotype x loneliness | Non-frailty | No Loneliness | Ref |  |
| Frailty phenotype x loneliness | Non-frailty | Loneliness | 1.13 (0.78-1.62) | 0.52 |
| Frailty phenotype x loneliness | Pre-frailty | No Loneliness | 1.16 (1.04-1.30) | 0.01 |
| Frailty phenotype x loneliness | Pre-frailty | Loneliness | 1.52 (1.23-1.89) | <0.001 |
| Frailty phenotype x loneliness | Frailty | No Loneliness | 1.72 (1.28-2.32) | <0.001 |
| Frailty phenotype x loneliness | Frailty | Loneliness | 1.85 (1.56-2.19) | <0.001 |
| Frailty phenotype x social isolation | Non-frailty | No Social isolation | Ref |  |
| Frailty phenotype x social isolation | Non-frailty | Social isolation | 1.08 (0.80-1.45) | 0.612 |
| Frailty phenotype x social isolation | Pre-frailty | No Social isolation | 1.18 (1.05-1.33) | 0.005 |
| Frailty phenotype x social isolation | Pre-frailty | Social isolation | 1.26 (1.03-1.53) | 0.025 |
| Frailty phenotype x social isolation | Frailty | No Social isolation | 1.70 (1.42-2.03) | <0.001 |
| Frailty phenotype x social isolation | Frailty | Social isolation | 2.28 (1.75-2.96) | <0.001 |

**Note:** Death before incident PAD was treated as a competing event. Models were adjusted for age, sex, ethnicity, Townsend Deprivation Index, educational attainment, smoking status, alcohol consumption, body mass index, mean arterial pressure, lipid-lowering medication, insulin use, aspirin use, antihypertensive medication, low-density lipoprotein cholesterol, glycated hemoglobin, estimated glomerular filtration rate, and dietary pattern. CI, confidence interval; eGFR, estimated glomerular filtration rate; HbA1c, glycated hemoglobin; LDL-C, low-density lipoprotein cholesterol; PAD, peripheral artery disease; sHR, subdistribution hazard ratio.

**Table S23. Associations of loneliness, social isolation, and frailty with incident peripheral artery disease after additional adjustment for the triglyceride–glucose index and collinearity diagnostics**

**Panel A. TyG-adjusted associations with incident PAD**

| **Exposure** | **Category** | **PAD cases/total, n (%)** | **HR** | **95% CI** | **P value** |
| --- | --- | --- | --- | --- | --- |
| **Loneliness** | | | | | |
|  | No loneliness | 1,662/20,594 (8.07) | Reference |  |  |
|  | Loneliness | 211/1,988 (10.61) | 1.25 | 1.08–1.44 | 0.003 |
|  | Per 1-point increase | 1,873/22,582 (8.29) | 1.15 | 1.07–1.23 | <0.001 |
| **Social isolation** | | | | | |
|  | No social isolation | 1,572/19,780 (7.95) | Reference |  |  |
|  | Social isolation | 301/2,802 (10.74) | 1.25 | 1.10–1.42 | 0.001 |
|  | Per 1-point increase | 1,873/22,582 (8.29) | 1.14 | 1.07–1.21 | <0.001 |
| **Frailty** | | | | | |
|  | Non-frail | 501/7,573 (6.62) | Reference |  |  |
|  | Pre-frail | 1,021/12,431 (8.21) | 1.22 | 1.09–1.36 | <0.001 |
|  | Frail | 351/2,578 (13.62) | 1.99 | 1.72–2.30 | <0.001 |
|  | Per 1-point increase | 1,873/22,582 (8.29) | 1.25 | 1.20–1.30 | <0.001 |

**Panel B. Collinearity diagnostics for the TyG-adjusted model**

| **Variable** | **GVIF** | **df** | **GVIF^(1/[2 × df])** |
| --- | --- | --- | --- |
| Age | 1.124 | 1 | 1.060 |
| Sex | 1.116 | 1 | 1.056 |
| Ethnicity | 1.083 | 1 | 1.041 |
| Townsend Deprivation Index | 1.152 | 1 | 1.073 |
| Educational attainment | 1.035 | 1 | 1.017 |
| Smoking status | 1.136 | 2 | 1.032 |
| Body mass index | 1.217 | 1 | 1.103 |
| Mean arterial pressure | 1.102 | 1 | 1.050 |
| Lipid-lowering medication | 1.465 | 1 | 1.210 |
| Antihypertensive medication | 1.254 | 1 | 1.120 |
| Insulin use | 1.062 | 1 | 1.030 |
| Aspirin use | 1.196 | 1 | 1.094 |
| Low-density lipoprotein cholesterol | 1.483 | 1 | 1.218 |
| Glycated hemoglobin | 1.007 | 1 | 1.003 |
| Healthy dietary pattern | 1.049 | 1 | 1.024 |
| Frailty score | 1.198 | 1 | 1.094 |
| TyG index | 1.088 | 1 | 1.043 |
| Social isolation score | 1.136 | 1 | 1.066 |
| Loneliness score | 1.117 | 1 | 1.057 |

**Note:** The TyG index could not be calculated for 11.8% of participants before imputation. Missing triglyceride and glucose values were imputed separately using multiple imputation by chained equations, and the TyG index was derived within each imputed dataset as ln [triglycerides (mg/dL) × glucose (mg/dL)/2]. The Cox models included the covariates in the primary multivariable-adjusted model, with the TyG index and fasting duration additionally included; estimates were pooled using Rubin’s rules. Panel B presents collinearity diagnostics from a model containing the three continuous exposure scores, the TyG index, and the listed covariates. All adjusted GVIF values were ≤1.22. CI, confidence interval; df, degrees of freedom; GVIF, generalized variance inflation factor; HR, hazard ratio; PAD, peripheral artery disease; TyG, triglyceride–glucose.

**Table S24. Multiple-imputation analysis of frailty, social disconnection, and incident peripheral artery disease**

| **Exposure** | **PAD cases/total, n (%)** | **Unadjusted HR (95% CI)** | **P value** | **Adjusted HR (95% CI)** | **P value** |
| --- | --- | --- | --- | --- | --- |
| **Loneliness** |  |  |  |  |  |
| No loneliness | 2,600/31,228 (8.33) | Reference |  | Reference |  |
| Loneliness | 336/3,176 (10.58) | 1.32 (1.18–1.48) | <0.001 | 1.21 (1.08–1.36) | 0.001 |
| Per 1-point increase | 2,936/34,404 (8.53) | 1.20 (1.14–1.27) | <0.001 | 1.15 (1.09–1.21) | <0.001 |
| **Social isolation** |  |  |  |  |  |
| No social isolation | 2,466/29,758 (8.29) | Reference |  | Reference |  |
| Social isolation | 470/4,646 (10.12) | 1.30 (1.17–1.43) | <0.001 | 1.15 (1.04–1.27) | 0.007 |
| Per 1-point increase | 2,936/34,404 (8.53) | 1.18 (1.12–1.24) | <0.001 | 1.11 (1.05–1.16) | <0.001 |
| **Frailty phenotype** |  |  |  |  |  |
| Non-frail | 775/11,662 (6.65) | Reference |  | Reference |  |
| Pre-frail | 1,654/19,062 (8.68) | 1.35 (1.24–1.47) | <0.001 | 1.30 (1.19–1.42) | <0.001 |
| Frail | 507/3,680 (13.78) | 2.37 (2.12–2.65) | <0.001 | 2.05 (1.82–2.30) | <0.001 |
| Per 1-point increase | 2,936/34,404 (8.53) | 1.30 (1.26–1.35) | <0.001 | 1.26 (1.21–1.30) | <0.001 |

**Note.** The analysis included 34,404 participants without prevalent PAD and 2,936 incident PAD events. Missing frailty and social-disconnection items were imputed using multiple imputation by chained equations; exposure scores and categories were reconstructed within each imputed dataset, and estimates were pooled using Rubin’s rules. The adjusted model included the same covariates as the primary analysis. CI, confidence interval; HR, hazard ratio; PAD, peripheral artery disease.

**Table S25. Multiple-imputation sensitivity analysis of the joint associations of frailty and social disconnection with incident peripheral artery disease**

| **Frailty status** | **Social-disconnection status** | **Adjusted HR (95% CI)** | **P value** |
| --- | --- | --- | --- |
| **Joint analysis with loneliness** | |  |  |
| Non-frail | No loneliness | Reference |  |
| Non-frail | Loneliness | 1.07 (0.80–1.43) | 0.663 |
| Pre-frail | No loneliness | 1.29 (1.18–1.41) | <0.001 |
| Pre-frail | Loneliness | 1.71 (1.46–2.00) | <0.001 |
| Frail | No loneliness | 2.00 (1.59–2.52) | <0.001 |
| Frail | Loneliness | 2.27 (1.91–2.56) | <0.001 |
| **Joint analysis with social isolation** | |  |  |
| Non-frail | No social isolation | Reference |  |
| Non-frail | Social isolation | 1.24 (1.00–1.54) | 0.056 |
| Pre-frail | No social isolation | 1.33 (1.21–1.46) | <0.001 |
| Pre-frail | Social isolation | 1.35 (1.17–1.57) | <0.001 |
| Frail | No social isolation | 2.00 (1.76–2.28) | <0.001 |
| Frail | Social isolation | 2.47 (2.03–3.00) | <0.001 |

**Note.** Hazard ratios were estimated in multivariable-adjusted Cox proportional hazards models after multiple imputation of frailty and social-disconnection items. The reference categories were non-frail participants without loneliness and non-frail participants without social isolation, respectively. CI, confidence interval; HR, hazard ratio; PAD, peripheral artery disease.

**Table S26. Associations of loneliness, social isolation, and frailty with incident peripheral artery disease in 2-year and 5-year lagged sensitivity analyses**

| **Exposure** | **Category** | **Cases/Total, n (%)** | **Unadjusted HR (95% CI)** | **P value** | **Multivariable-adjusted HR (95% CI)** | **P value** |
| --- | --- | --- | --- | --- | --- | --- |
| **A. Two-year lag analysis** | | | | | | |
| Loneliness | No loneliness | 1,514/20,235 (7.48) | Reference |  | Reference |  |
|  | Loneliness | 187/1,942 (9.63) | 1.33 (1.14-1.55) | <0.001 | 1.23 (1.05-1.43) | 0.009 |
| Loneliness score | Per 1-point increase | 1,701/22,177 (7.67) | 1.21 (1.13-1.30) | <0.001 | 1.15 (1.07-1.23) | <0.001 |
| Social isolation | No social isolation | 1,434/19,451 (7.37) | Reference |  | Reference |  |
|  | Social isolation | 267/2,726 (9.79) | 1.40 (1.23-1.60) | <0.001 | 1.24 (1.08-1.41) | 0.002 |
| Social isolation score | Per 1-point increase | 1,701/22,177 (7.67) | 1.19 (1.12-1.27) | <0.001 | 1.12 (1.05-1.20) | 0.001 |
| Frailty phenotype | Non-frail | 474/7,483 (6.33) | Reference |  | Reference |  |
|  | Pre-frail | 926/12,231 (7.57) | 1.23 (1.10-1.37) | <0.001 | 1.17 (1.05-1.31) | 0.006 |
|  | Frail | 301/2,463 (12.22) | 2.17 (1.88-2.51) | <0.001 | 1.85 (1.59-2.16) | <0.001 |
| Frailty score | Per 1-point increase | 1,701/22,177 (7.67) | 1.28 (1.23-1.33) | <0.001 | 1.23 (1.18-1.28) | <0.001 |
| **B. Five-year lag analysis** | | | | | | |
| Loneliness | No loneliness | 1,229/19,486 (6.31) | Reference |  | Reference |  |
|  | Loneliness | 150/1,841 (8.15) | 1.32 (1.11-1.56) | 0.001 | 1.23 (1.04-1.46) | 0.019 |
| Loneliness score | Per 1-point increase | 1,379/21,327 (6.47) | 1.19 (1.10-1.28) | <0.001 | 1.13 (1.05-1.23) | 0.002 |
| Social isolation | No social isolation | 1,171/18,771 (6.24) | Reference |  | Reference |  |
|  | Social isolation | 208/2,556 (8.14) | 1.35 (1.17-1.57) | <0.001 | 1.21 (1.04-1.41) | 0.013 |
| Social isolation score | Per 1-point increase | 1,379/21,327 (6.47) | 1.18 (1.10-1.27) | <0.001 | 1.12 (1.04-1.21) | 0.002 |
| Frailty phenotype | Non-frail | 400/7,271 (5.50) | Reference |  | Reference |  |
|  | Pre-frail | 753/11,776 (6.39) | 1.19 (1.05-1.34) | 0.005 | 1.13 (1.00-1.28) | 0.056 |
|  | Frail | 226/2,280 (9.91) | 1.98 (1.68-2.33) | <0.001 | 1.68 (1.41-2.00) | <0.001 |
| Frailty score | Per 1-point increase | 1,379/21,327 (6.47) | 1.24 (1.19-1.30) | <0.001 | 1.19 (1.14-1.25) | <0.001 |

**Note:** In the 2-year and 5-year lag analyses, participants who developed PAD or were censored before the corresponding landmark were excluded, and follow-up began at 2 or 5 years after baseline. The multivariable-adjusted models included age, sex, ethnicity, Townsend Deprivation Index, educational attainment, smoking status, alcohol consumption, body mass index, mean arterial pressure, lipid-lowering medication, insulin use, aspirin use, antihypertensive medication, low-density lipoprotein cholesterol, glycated hemoglobin, estimated glomerular filtration rate, and dietary pattern. CI, confidence interval; HR, hazard ratio; PAD, peripheral artery disease.

**Figure S1. Assumed causal diagram for the exploratory mediation analysis.**

Measured baseline covariates were assumed to affect the exposure, corresponding metabolomic risk score, and incident peripheral artery disease. The exposure was assumed to affect incident peripheral artery disease both directly and indirectly through the corresponding metabolomic risk score. MRS, metabolomic risk score; PAD, peripheral artery disease.

**Figure S2. LASSO-Cox selection of metabolites for the exposure-specific metabolomic risk scores in the training set**

Panels A–C show the selection procedures for the frailty-, loneliness-, and social isolation-related metabolomic risk scores, respectively. For each score, the left panel shows the 10-fold cross-validation curve of the Cox partial-likelihood deviance against log-transformed λ. The blue and red dashed lines indicate the values of λ giving the minimum cross-validation error and the largest λ within one standard error of the minimum, respectively. The right panel shows the coefficient trajectories as λ varies. Metabolites with nonzero coefficients at the one-standard-error λ were retained. LASSO, least absolute shrinkage and selection operator; MRS, metabolomic risk score.

**Figure S3. Time-dependent receiver operating characteristic curves for the exposure-specific metabolomic risk scores in the training set**

Rows A–C show the frailty-, loneliness-, and social isolation-related metabolomic risk scores, respectively. The columns show the time-dependent receiver operating characteristic curves for incident peripheral artery disease at 5, 10, and 15 years. Solid lines represent the receiver operating characteristic curves, shaded areas represent 95% confidence intervals, and diagonal dashed lines indicate discrimination no better than chance. The area under the curve and its 95% confidence interval are displayed in each panel. AUC, area under the curve; CI, confidence interval; MRS, metabolomic risk score; PAD, peripheral artery disease.

**Figure S4. Time-dependent receiver operating characteristic curves for the exposure-specific metabolomic risk scores in the holdout validation set**

Rows A–C show the frailty-, loneliness-, and social isolation-related metabolomic risk scores, respectively. The scores were calculated using the metabolites and coefficients fixed in the training set. The columns show the time-dependent receiver operating characteristic curves for incident peripheral artery disease at 5, 10, and 15 years. Solid lines represent the receiver operating characteristic curves, shaded areas represent 95% confidence intervals, and diagonal dashed lines indicate discrimination no better than chance. The area under the curve and its 95% confidence interval are displayed in each panel. AUC, area under the curve; CI, confidence interval; MRS, metabolomic risk score; PAD, peripheral artery disease.

**Figure S5. SHAP main and interaction effects in the MRS-based exploratory XGBoost model**

The figure compares the mean absolute SHAP main effect of each feature with the sum of its mean absolute pairwise SHAP interaction effects in the exploratory XGBoost model for 10-year peripheral artery disease status. Blue bars represent main effects, and orange bars represent summed interaction effects involving each feature. Larger values indicate a greater contribution to the fitted model output. Age, sex, and ethnicity were excluded by design. SHAP values describe feature attribution within the fitted model and do not establish incremental predictive value or causality. BMI, body mass index; eGFR, estimated glomerular filtration rate; HbA1c, glycated hemoglobin; LDL-C, low-density lipoprotein cholesterol; MAP, mean arterial pressure; MRS, metabolomic risk score; PAD, peripheral artery disease; PRS, polygenic risk score; SHAP, Shapley additive explanations; TDI, Townsend Deprivation Index; XGBoost, extreme gradient boosting.

**Figure S6. Pairwise SHAP interaction matrix for the MRS-based exploratory XGBoost model**

The matrix shows pairwise SHAP interactions among variables included in the MRS-based exploratory XGBoost model for 10-year peripheral artery disease status. The lower triangle presents the mean absolute SHAP interaction strength for each variable pair, with numerical values shown within the cells. The upper triangle shows the distributions of signed SHAP interaction values, while diagonal panels show feature-specific main effects. Point colors indicate the underlying feature values, ranging from low (blue) to high (orange). Age, sex, and ethnicity were excluded by design. BMI, body mass index; eGFR, estimated glomerular filtration rate; HbA1c, glycated hemoglobin; LDL-C, low-density lipoprotein cholesterol; MAP, mean arterial pressure; MRS, metabolomic risk score; PAD, peripheral artery disease; PRS, polygenic risk score; SHAP, Shapley additive explanations; TDI, Townsend Deprivation Index; XGBoost, extreme gradient boosting.

**Figure S7. SHAP main and interaction effects in the phenotype-based exploratory XGBoost model**

The figure compares the mean absolute SHAP main effect of each feature with the sum of its mean absolute pairwise SHAP interaction effects in the phenotype-based exploratory XGBoost model for 10-year peripheral artery disease status. Blue bars represent main effects, and orange bars represent summed interaction effects involving each feature. The model included frailty, loneliness, and social isolation scores together with conventional clinical, lifestyle, and genetic variables. Age, sex, and ethnicity were excluded by design. Larger values indicate a greater contribution to the fitted model output but do not establish incremental predictive value or causality. BMI, body mass index; eGFR, estimated glomerular filtration rate; HbA1c, glycated hemoglobin; LDL-C, low-density lipoprotein cholesterol; MAP, mean arterial pressure; PAD, peripheral artery disease; PRS, polygenic risk score; SHAP, Shapley additive explanations; TDI, Townsend Deprivation Index; XGBoost, extreme gradient boosting.

**Figure S8. Pairwise SHAP interaction matrix for the phenotype-based exploratory XGBoost model**

The matrix shows pairwise SHAP interactions among variables included in the phenotype-based exploratory XGBoost model for 10-year peripheral artery disease status. The lower triangle presents the mean absolute SHAP interaction strength for each variable pair, with numerical values shown within the cells. The upper triangle shows the distributions of signed SHAP interaction values, while diagonal panels show feature-specific main effects. Point colors indicate the underlying feature values, ranging from low (blue) to high (orange). The model included frailty, loneliness, and social isolation scores, whereas age, sex, and ethnicity were excluded by design. BMI, body mass index; eGFR, estimated glomerular filtration rate; HbA1c, glycated hemoglobin; LDL-C, low-density lipoprotein cholesterol; MAP, mean arterial pressure; PAD, peripheral artery disease; PRS, polygenic risk score; SHAP, Shapley additive explanations; TDI, Townsend Deprivation Index; XGBoost, extreme gradient boosting.
